# Protocol for a cohort study of comprehensive intervention of psoriasis and diabetes comorbidity prevention and control using traditional Chinese medicine of blood-activating and stasis-dissolving method

**DOI:** 10.3389/fmed.2026.1831495

**Published:** 2026-10-14

**Authors:** Qingyun Wang, Jingwen Yeap, Pengbo Gao, Jiale Chen, Jiao Wang, Liu Liu, Jingjie He, Xiaoying Sun, Xin Li

**Affiliations:** 1 Department of Dermatology, Yueyang Hospital of Integrated Traditional Chinese and Western Medicine, Shanghai University of Traditional Chinese Medicine, Shanghai, China; 2 Institute of Dermatology, Shanghai Academy of Traditional Chinese Medicine, Shanghai, China

**Keywords:** biomarkers, blood-activating and stasis-dissolving method, diabetes mellitus, insulin resistance, metabolic diseases, psoriasis, Taodan granules

## Abstract

**Background:**

Psoriasis is a chronic systemic inflammatory disease associated with an increased risk of metabolic dysfunction, including type 2 diabetes mellitus (T2DM). Insulin resistance and chronic inflammation may contribute to this association. Blood-activating and stasis-dissolving therapies in traditional Chinese medicine (TCM) have shown potential anti-inflammatory and metabolic benefits. This study aimed to evaluate an integrated TCM-based intervention in patients with psoriasis at high risk of T2DM.

**Methods:**

Participants aged 18–80 who met predefined diagnostic criteria for psoriasis vulgaris and blood-stasis syndrome will be recruited. Individuals identified as being at high risk for T2DM using a validated multifactorial diagnostic model will be allocated to two groups according to clinical assessment and treatment preference. The conventional strategy group (CSG) will receive guideline-based conventional psoriasis treatment, while the integrated strategy group (ISG) will receive standard care combined with Taodan granules (TDG), fish oil supplementation, and a structured aerobic exercise programme. The primary analysis will employ linear mixed-effects models with a difference-in-differences approach to compare changes in outcomes between groups. Propensity score matching will be performed as a sensitivity analysis to balance baseline characteristics.

**Outcomes:**

The primary outcome is the change in insulin resistance from baseline to Week 16, assessed using the homeostasis model assessment of insulin resistance (HOMA-IR). Key secondary outcomes include changes in psoriasis severity assessed by the psoriasis area and severity index (PASI), including PASI 50, PASI 75, and PASI 90 response rates. Exploratory outcomes include the incidence of new-onset T2DM and changes in circulating inflammatory biomarkers, including IL-1β, IL-6, TNF-α, IL-17A, and FGL1, to explore potential mechanisms underlying the effects of the integrated intervention.

**Conclusion:**

This study aims to evaluate the effects of an integrated TCM-based intervention on dermatological and metabolic outcomes in patients with psoriasis at high risk of T2DM. The findings may provide clinical evidence to support policymakers in developing management strategies for psoriasis and related metabolic comorbidities.

**Trial registration:**

This trial was registered at ClinicalTrials.gov (ID: NCT06402760).

## 1 Introduction

Psoriasis, an immune-mediated chronic inflammatory skin disease characterized by elevated erythematous plaques covered with silver scale, is often accompanied by pruritus. Recent systematic reviews estimate that the global prevalence of psoriasis can be approximately 2% to 3%, with geographic variation ranging from 0.5% in parts of Asia to 11.4% in northern Europe (1, 2). In China, large-scale epidemiological surveys have reported a prevalence of approximately 0.47% (3). The pathogenesis of psoriasis is multifaceted and encompasses genetic predispositions, immune system irregularities, and environmental factors that work together to trigger persistent inflammation. This inflammation, in turn, leads to abnormal proliferation of keratinocytes and impaired cellular differentiation, ultimately manifesting clinical features of the disease (4). Psoriasis remains incurable and exerts severe adverse impacts on patients' quality of life. Nevertheless, several treatment strategies including topical therapy, phototherapy, oral systemic therapy, and biologics can relieve the related skin lesions (5). Beyond its cutaneous manifestations, psoriasis is increasingly recognized as a systemic disorder. Chronic systemic inflammation in psoriasis is a well-established driver of metabolic dysfunction, significantly increasing the risk of comorbidities such as cardiovascular disease (6), obesity (7), dementia (8), inflammatory bowel disease (9), and type 2 diabetes mellitus (T2DM) (10).

T2DM is characterized by insulin resistance and relative insulin deficiency, resulting in hyperglycaemia (11). It can lead to acute life-threatening complications such as diabetic ketoacidosis and hypoglycaemic coma, as well as chronic vascular complications such as cardiovascular disease and diabetic nephropathy (12). Epidemiological studies indicate a profound bidirectional association between psoriasis and T2DM: T2DM elevates psoriasis risk by 40%, while psoriasis increases T2DM risk by 38%, with this association being more displayed in patients aged ≥ 50 (13). Moreover, the risk exhibits a dose-dependent pattern: mild psoriasis increases the T2DM risk by 21%, while severe psoriasis increases it by 64%. In addition, for every 10% increase in Body Surface Area (BSA) affected by psoriasis, the risk of developing T2DM increases by an additional 19.3% (14). These epidemiological findings support risk stratification for T2DM among patients with psoriasis. Accordingly, we developed and validated a multifactorial diagnostic model in the past (15). In the present study, this established diagnostic model will be used to identify a high-risk cohort and evaluate whether the integrated intervention can mitigate early metabolic deterioration. Taodan granules (TDG), a formula developed based on the TCM principle of “blood-activating and stasis-dissolving”, has been used in clinical practice to treat psoriasis vulgaris with blood stasis syndrome (16). According to TCM theory, “blood stasis” is a consistent pathological feature throughout the progression of psoriasis; as the tangible stasis persists, it leads to consumption of qi, blood and body fluids, ultimately contributing to the development of T2DM (known in TCM as “Xiao Ke”) (17). Both psoriasis and T2DM share a common pathological link: blood stasis. Therefore, our hypothesis is that TDG can reduce the risk of T2DM by resolving blood stasis and improving systemic microcirculation. This aligns with the principle of “treating different diseases with the same method” (Yi Bing Tong Zhi).

Guided by the core therapeutic principle of “blood-activating and stasis-dissolving,” we developed a comprehensive intervention strategy. Our previous meta-analyses showed that lifestyle interventions may also be beneficial, including fish oil supplementation which improved PASI scores and lipid profiles (18), and moderate-intensity exercise (≥150 min/week), which was associated with reduced systemic inflammation (19). Therefore, this study is designed to provide prospective evidence that an integrated TCM-based intervention may improve insulin resistance and dermatological outcomes in high-risk patients with psoriasis. By evaluating these parameters, we aim to explore the potential feasibility of combining lifestyle interventions with TDG therapy to address metabolic and dermatological problems in this cohort.

## 2 Methods

### 2.1 Design

This is a prospective, two-arm, patient preference-based, non-randomized cohort study. This study will investigate the preventive and therapeutic effects of a comprehensive intervention comprising TDG, fish oil, and aerobic exercise in patients with psoriasis who are at high risk of comorbid T2DM. The trial will include three phases: a 1-week screening period, a 16-week treatment phase, and a 24-week follow-up period. After the 16-week treatment phase ends, participants in the ISG will discontinue TDG but will continue fish oil supplementation and the prescribed exercise regimen through Week 40; participants in the CSG will not receive fish oil supplementation or the structured exercise intervention at any stage. The Week 40 assessment will evaluate clinical and metabolic outcomes after discontinuation of TDG, while fish oil supplementation and the prescribed exercise regimen will continue. Participants will be recruited from the outpatient clinics and inpatient wards of the Department of Dermatology at the Yueyang Hospital of Integrated Traditional Chinese and Western Medicine, Shanghai University of Traditional Chinese Medicine. All study procedures were approved by the Ethics Committee of Yueyang Hospital of Integrated Traditional Chinese and Western Medicine, Shanghai University of Traditional Chinese Medicine (approval no. 2024-034) and will be conducted in accordance with the principles of the Declaration of Helsinki. All participants will receive comprehensive information about the study objectives and procedures, and provide written informed consent before being enrolled (Supplementary File 1). Patients and the public will not be involved in the design, implementation, report, or dissemination plans of this research.

During the screening phase, clinical data will be collected based on psoriasis and diabetes diagnostic models. Patients whose risk scores exceed the predefined threshold and who have no prior diagnosis of diabetes will undergo vital sign monitoring and laboratory testing, including complete blood count, liver and kidney function, glucose metabolism parameters [glycated hemoglobin (HbA1c), fasting plasma glucose, 2-h postprandial glucose, fasting insulin (FINS), and C-peptide], and lipid metabolism parameters [serum triglycerides (TG), total cholesterol (TC), high-density lipoprotein (HDL) cholesterol, low-density lipoprotein (LDL) cholesterol, apolipoprotein AI (ApoA-I), apolipoprotein B (ApoB), lipoprotein(a), and small dense low-density lipoprotein (sdLDL)]. Peripheral blood samples will also be collected to assess inflammatory and metabolic status by measuring serum concentrations of high-sensitivity C-reactive protein (hsCRP), interleukin-1β (IL-1β), interleukin-6 (IL-6), tumor necrosis factor-alpha (TNF-α), interleukin-17A (IL-17A), and fibrinogen-like protein 1 (FGL1). Eligible participants will then be enrolled into either the conventional strategy group (CSG) or the integrated strategy group (ISG) (emphasizing blood-activating and stasis-dissolving) based on a combination of the clinician's comprehensive assessment and the patient's individual preference. Psoriasis severity and indicators of glucose and lipid metabolism will be assessed at Week 16 and Week 40.

The trial was registered at ClinicalTrials.gov (NCT06402760; May 7, 2024). The data will be available at https://clinicaltrials.gov/study/NCT06402760. The participant flow is shown in Figure 1.

**Figure 1:**
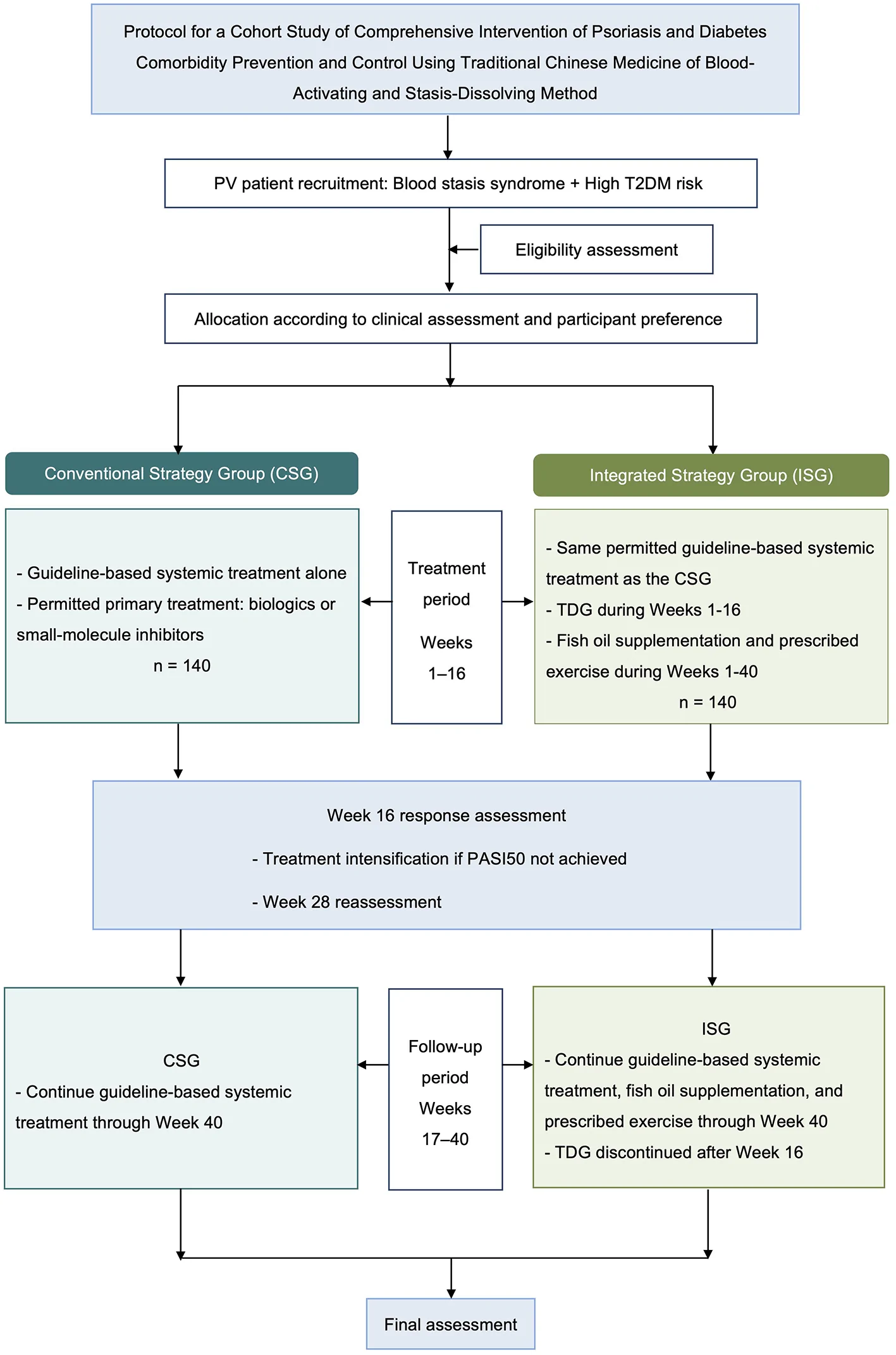
Flow chart of the study protocol. CSG, conventional strategy group; ISG, integrated strategy group; PASI, psoriasis area and severity index; PV, psoriasis vulgaris; T2DM, type 2 diabetes mellitus; TDG, Taodan granules.

### 2.2 Eligibility criteria

#### 2.2.1 Diagnostic criteria

The diagnostic criteria for psoriasis and blood-stasis syndrome type follow the Guideline for the Diagnosis and Treatment of Psoriasis in China (2023 Edition) (20). Diabetes will be diagnosed according to the “Chinese Guidelines for the Prevention and Treatment of T2DM (2020 Edition),” which requires the presence of classic symptoms (polyuria, polydipsia, polyphagia, and unexplained weight loss) plus at least one of the following: random blood glucose ≥ 11.1 mmol/L, fasting blood glucose ≥ 7.0 mmol/L, 2-h postprandial blood glucose ≥ 11.1 mmol/L during an oral glucose tolerance test, or HbA1c ≥ 6.5% (21). Insulin resistance will be assessed according to the “Expert Guidance on Methods and Applications for Insulin Resistance Assessment” using the quantitative insulin sensitivity check index (QUICKI), which will be calculated as QUICKI = 1/[log_10_(FPG, mg/dL) + log_10_(FINS, μU/mL)] (22). For the calculation of QUICKI, fasting plasma glucose and fasting insulin concentrations will be expressed in mg/dL and μU/mL, respectively, with QUICKI values ≤ 0.339 indicating insulin resistance.

#### 2.2.2 Inclusion criteria

The study inclusion criteria will be as follows: (1) participants must meet the diagnostic criteria for psoriasis vulgaris with blood-stasis syndrome, as defined by the Guideline for the Diagnosis and Treatment of Psoriasis in China (2023 Edition), with details of the clinical features and diagnostic procedures in Section 2.2.2.1; (2) a diagnosis of psoriasis vulgaris, regardless of whether the condition is newly diagnosed or previously established, with no restrictions on prior treatment among participants with pre-existing disease; (3) an elevated risk of comorbid diabetes mellitus as identified by the risk modeling (details are provided in Section 2.2.2.2); (4) age 18–80 years; and (5) provision of written informed consent for study participation.

##### 2.2.2.1 Diagnostic standardization

The diagnosis of TCM blood-stasis syndrome will be standardized using predefined clinical markers and independent assessment to improve the objectivity of syndrome differentiation. The diagnostic criteria will require predominantly dark-red or dull-purple plaques with thick, adherent scales, accompanied by systemic manifestations such as a dark-purple tongue body or sublingual varicosities. All diagnostic assessments will be performed independently by two certified TCM dermatologists, each with more than five years of clinical experience. To further improve diagnostic accuracy and minimize inter-observer variability, disagreements between the two primary assessors will be resolved by consensus or adjudication by a third senior consultant with more than 15 years of professional experience.

##### 2.2.2.2 Participant screening and risk stratification

Participants will be screened and stratified using a previously validated multifactorial diagnostic model (15). This model incorporates 11 clinical predictors: age, body mass index (BMI), marital status, drug allergy, tumor history, joint swelling and pain, nail pitting, dactylitis, duration of psoriasis, nail involvement, and PASI scores.

The model demonstrated high diagnostic accuracy with an Area Under the Curve of 0.875 (95% CI: 0.862–0.889) and was well-calibrated (Hosmer–Lemeshow test, *p* = 0.060; mean absolute error = 0.003). Based on the Youden Index, the optimal risk threshold was established at 0.016, yielding a sensitivity of 90.9% and a specificity of 69.2%. This threshold was chosen to ensure robust sensitivity for the early identification of T2DM risk in a clinical setting. Participants with a calculated predicted probability exceeding 0.016 will be classified as the high-risk cohort and invited to enroll in the study.

#### 2.2.3 Exclusion criteria

The exclusion criteria will be as follows: (1) diagnosis of T2DM; (12) concurrent progressive skin diseases that may interfere with the condition assessment; (3) severe or uncontrolled local or systemic infection (acute or chronic); (4) significant systemic illnesses, hematological abnormalities (hemoglobin, platelet count, white blood cell count, or absolute neutrophil count below the lower limit of normal), elevated alanine aminotransferase or aspartate aminotransferase (exceeding three times the upper limit of normal), total bilirubin exceeding 1.5 times the upper limit of normal, serum creatinine exceeding 1.5 times the upper limit of normal, or other clinically relevant laboratory anomalies; (5) history of malignant neoplasms, primary or secondary immune deficiency, and/or a hypersensitivity reaction; (6) major surgery within 8 weeks before study initiation or scheduled during the study period; (7) pregnancy or lactation; (8) history of alcohol abuse or drug abuse; (9) personal history of a serious mental illness; and (10) any other factors the researcher considers inappropriate for an individual's participation.

### 2.3 Interventions

Patients in the CSG will receive tiered, guideline-based conventional treatment tailored to disease severity under a pragmatic design. To balance real-world applicability with analytical rigor, the protocol restricts conventional treatment options to currently recommended systemic therapies commonly used for moderate-to-severe psoriasis. Permitted primary treatments include biologics (e.g., adalimumab, secukinumab, and ustekinumab) and small-molecule inhibitors (e.g., apremilast and deucravacitinib). The attending physician will select the treatment according to the participant's clinical condition and the Guideline for the Diagnosis and Treatment of Psoriasis in China (2023 Edition). Topical therapies, phototherapy, and other conventional systemic agents, such as methotrexate and cyclosporine, are not permitted as primary treatments within the study.

Treatment response will be assessed using the PASI. Participants who have not achieved PASI 50 at the Week 16 visit may undergo treatment intensification through switching to another permitted therapy within the same therapeutic class, in accordance with the applicable prescribing information and the Guideline for the Diagnosis and Treatment of Psoriasis in China (2023 Edition). The intensified regimen will be maintained from Week 16 until the Week 28 reassessment, unless a change is required for safety or another clinically compelling reason. Response to treatment intensification will be formally reassessed at the Week 28 visit. Participants who still have not achieved PASI 50 may receive protocol-permitted topical rescue therapy, including high-potency topical corticosteroids, calcipotriol or other topical vitamin D analogs, 3% salicylic acid, and topical retinoids such as tazarotene. Oral antihistamines are permitted at any time during the study for participants with severe pruritus. Protocol-permitted topical rescue therapy may continue through Week 40 when clinically required.

Phototherapy and non-permitted systemic therapies, including systemic corticosteroids, systemic retinoids such as acitretin, cyclosporine, and methotrexate, are not permitted under the study treatment strategy. Any medically necessary use of a non-permitted treatment will be recorded with the treatment name, dose, timing, duration, and reason for use and will be classified and analyzed in accordance with Sections 2.8 and 2.9 and Supplementary File 4. All treatment intensifications, switches, dose or frequency changes, rescue therapies, and non-permitted treatments will be recorded in real time in the “Medical Road Cloud” system.

The ISG will receive the following additional interventions alongside the standard care:

(1) TDG, a Chinese herbal formula based on the principle of blood-activating and stasis-dissolving, is manufactured by Sichuan Neo-green Pharmaceutical Technology Development Co., Ltd. for oral administration. The formula composition is detailed in Table 1.

**Table 1:** Ingredients of Taodan granules.

Chinese pinyin	Genus species (latin name)	Plant part	Amounts
Tao Ren	*Prunus persica* (L.) Batsch	Seed	10 g
Dan Shen	*Salvia miltiorrhiza* Bunge	Root	30 g
Chuan Xiong	*Ligusticum wallichii*	Rhizome	10 g
E Zhu	*Curcuma zedoaria* (Christm.) Rosc.	Rhizome	30 g
Huang Qi	*Astragalus adsurgens* Pall.	Root	15 g
Dang Gui	*Angelica sinensis (Oliv.) Diels*	Root and rhizome	15 g
Chuan Niu Xi	*Achyranthes bidentata Blume*	Root	15 g
Ba Qia	*Smilax china L*.	Rhizome	30 g
Gan Cao	*G. uralensis*	Root	10 g

All individual herb granules are sourced from GMP-certified suppliers: most are produced according to national standards, while E Zhu and Ba Qia, for which no national standards are currently available, comply with manufacturer's enterprise quality standards approved by the regulatory authorities. The manufacturing site holds quality management system certifications from the China Quality Certification Center (2020, 2023). All quality control procedures follow the requirements of the Chinese Pharmacopeia (2020 edition). Thin-layer chromatography (TLC) is used for qualitative identification, and high-performance liquid chromatography (HPLC) for quantitative analysis. Quantitative HPLC focuses on the marker compounds of the two principal (‘monarch') herbs in the formula: salvianolic acid B in Salvia miltiorrhiza (acceptance range: 28.0–59.0 mg/g) and amygdalin in Prunus persica (acceptance range: 20.0–50.0 mg/g), consistent with the national herbal granule standards (YBZ-PFKL series). Contaminant testing, including pesticide residues, heavy metals (Pb, Cd, As, Hg, Cu), sulfur dioxide (SO_2_), and aflatoxins, is performed using validated methods (e.g., UPLC-ICP-MS, GC-MS/MS). All test results are within permissible limits. During formulation preparation, the required amount of each herb granule (according to the daily dose listed in Table 1) is accurately weighed and physically blended to obtain a homogeneous mixture. The blended granules are then packaged into boxes, each containing seven compartments. Each compartment contains a single dose (half of the daily dose). Boxes are dispensed every 4 weeks. To monitor treatment adherence, patients are required to return used medication packaging at each visit before receiving the next 4-week supply. Adherence to TDG will be quantified by counting returned sachets. Each daily dose consists of two compartments, administered as one compartment in the morning and one compartment in the evening after meals. For each administration, one compartment is dissolved in approximately 50 mL of warm water, stirred until mostly dissolved, then diluted with another 100 mL of hot water before drinking.

(2) Participants in the ISG will purchase fish oil capsules themselves and take 1 g twice daily, in the morning and evening with meals, from baseline through Week 40 under the guidance of the project team. Participants will be instructed to select products meeting the prespecified minimum requirements for EPA/DHA content, formulation, purity, packaging, and storage, as detailed in Supplementary File 2. Fish oil adherence will be assessed from participant records and the returned original packaging, remaining capsules, and product information reviewed at each 4-week visit. Adherence during Weeks 1–16 will be considered adequate when the participant has taken at least 80% of the scheduled fish oil doses. Fish oil use and adherence monitoring will continue during Weeks 17–40, including review at Week 20, 24, 28, 32, 36, and 40 visits. Product name, manufacturer, declared EPA/DHA content, batch number, expiry date, dose, treatment interruptions, and potentially related adverse events will be recorded.

(3) Participants in the ISG will follow a standardized moderate-intensity aerobic exercise programme based on the frequency, intensity, time, and type (FITT) framework from baseline through Week 40. The prescribed target is at least 150 min of moderate-intensity aerobic exercise per week, with a target heart rate of approximately 60%−70% of the age-predicted maximum heart rate (MHR = 220 – age). Recommended activities include brisk walking, cycling, low-impact aerobics, and stepping exercises. For participants with psoriatic arthritis or other physical limitations, the study physician may prescribe an appropriate lower-impact alternative, such as Baduanjin, Wuqinxi, Yijinjing, or Tai Chi (36–40).

During Weeks 1–4, participants will gradually progress toward the full weekly target according to an individualized ramp-up plan. From Week 5 through Week 40, participants will continue the prescribed weekly target. Exercise will be recorded using the participant diary and ACVOR wearable-device records. A week will be classified as adherent when the participant completes the prescribed weekly exercise target at the required moderate intensity; during Weeks 1–4, adherence will be assessed against the participant's individualized weekly ramp-up target. Exercise adherence during Weeks 1–16 will be considered adequate when at least 80% of the prescribed weeks are classified as adherent. Post-baseline exercise exposure will be summarized by group and may be examined in exploratory analyses but will not be included as a continuous adjustment covariate in the primary confirmatory model.

The intervention schedule differs across components. TDG is administered only during Weeks 1–16. Fish oil supplementation and the prescribed exercise begin at baseline and continue through Week 40 for the ISG. Adherence to fish oil supplementation and exercise will continue to be monitored at the Week 20, 24, 28, 32, 36, and 40 visits. Fish oil adherence will be assessed using the participant's weekly records and returned product packaging, including remaining capsules, whereas exercise adherence will be assessed using the weekly diary and ACVOR wearable-device records. Detailed procedures for intervention delivery, monitoring, missed sessions, and adherence assessment are provided in Supplementary File 2.

### 2.4 Laboratory procedures

All participants are required to fast for at least 8 hours (overnight fast, only small amounts of water allowed) before blood draw, which is uniformly performed at 8:00 AM in the clinical laboratory of Yueyang Hospital. Following collection of fasting venous blood, samples for FINS, C-peptide, liver function, kidney function, and fasting plasma glucose are collected in serum separator tubes containing clot activator, and complete blood count, HbA1c, and hsCRP are collected in EDTA tubes. After the fasting draw, participants consume a standard breakfast, and a second venous blood sample is taken 2 h later for 2-h postprandial glucose (collected in sodium fluoride tubes). All routine laboratory tests (including complete blood count, HbA1c, hsCRP, FINS, C-peptide, fasting plasma glucose, 2-h postprandial glucose, liver function, kidney function, and the lipid panel TG, TC, HDL-C, LDL-C, ApoA-I, ApoB, lipoprotein(a), and sdLDL) are processed within 2 hours after collection following the hospital's standard operating procedures.

Serum levels of IL-1β, IL-6, TNF-α, IL-17A, and FGL1 will be quantified using commercial sandwich enzyme-linked immunosorbent assay (ELISA) kits (supplier and catalog numbers to be confirmed and documented prior to assay commencement) on an HRP-TMB colourimetric platform, with absorbance read at 450 nm. Whole blood will be collected into plain tubes, kept upright at 4 °C for 60 min to clot, centrifuged at 4 °C and 3000 rpm for 15 min, and the serum will then be immediately aliquoted and stored at −80 °C, avoiding repeated freeze-thaw cycles. All samples will be assayed in a single batch after collection to minimize inter-batch variability. Each sample and standard will be measured in duplicate. A four-parameter logistic standard curve will be generated per batch using kit-supplied calibrators, with a minimum acceptable *R*^2^ ≥ 0.99; batches failing this criterion will be rejected and re-run.

Assay performance will be accepted only if intra-assay coefficient of variation (CV) < 10%, inter-assay CV < 15%, recovery 80%−120%, and linearity *R*^2^ ≥ 0.99. The lower limit of detection (LOD) and lower limit of quantification (LLOQ) for each analyte will be obtained from the manufacturer and documented; values below the LLOQ will be assigned LLOQ/2 for statistical analysis. Additionally, each assay batch must include a full standard curve; internal quality control samples must fall within 80%−120% recovery; otherwise, the batch will be invalidated and the affected samples re-tested using a single thawed aliquot from the stored serum. Samples from the same participant across all time points will be assayed within the same batch whenever possible. Components from different kit lots will not be mixed, and all lot numbers will be recorded. Results will be re-tested if they exceed the laboratory reference range by >three-fold, are clinically inconsistent with the participant's status, or if batch-level quality control fails. Any persistently unexplained results after re-testing will be reviewed and documented by two certified laboratory physicians, with consultation of the principal investigator (PI) as needed.

For each exploratory biomarker assay, the final trial report will provide the manufacturer, catalog number, lot number, limit of detection, lower limit of quantification, intra-assay coefficient of variation, inter-assay coefficient of variation, and all prespecified quality-control results.

### 2.5 Outcomes

#### 2.5.1 Primary outcome

The primary outcome is the change in insulin resistance from baseline to Week 16, as measured by the homeostasis model assessment for insulin resistance (HOMA-IR). This index is calculated using the formula: HOMA-IR = [fasting plasma glucose (FPG, mmol/L) × fasting insulin (FINS, mIU/L)]/22.5. It serves as the central metric to evaluate the intervention's impact on systemic metabolic health. Note that FPG and FINS units differ between QUICKI (mg/dL, μU/mL) and HOMA-IR (mmol/L, mIU/L) calculations, consistent with each index's standard formula.

#### 2.5.2 Key secondary outcomes

Clinical efficacy will be evaluated by the change in the psoriasis area and severity index (PASI) score from baseline. The PASI score will be assessed every 4 weeks throughout the 40-week study period to evaluate longitudinal clinical responses. Categorical clinical response rates will be quantified at Week 16 by the proportion of participants achieving PASI 50, PASI 75, and PASI 90. Additionally, the recurrence rate of psoriasis lesions will be monitored during the post-treatment follow-up period to evaluate the durability of the therapeutic effect.

#### 2.5.3 Secondary outcomes

Secondary outcomes will include the change in HOMA-IR from baseline to Week 40, changes in lipid parameters (serum TG, TC, HDL-C, LDL-C, ApoA-I, ApoB, lipoprotein(a), and sdLDL), and changes in psoriasis severity indices, including BSA, PGA, DLQI, pruritus VAS, and the TCM symptom score for blood-stasis syndrome. These outcomes will be assessed at Weeks 16 and 40.

The indicators of blood-stasis syndrome are presented in Table 2. The total score ranges from 0 to 6, with higher scores indicating more severe blood-stasis syndrome. In the absence of a psoriasis-specific cut-off, a score of ≥3 is used to define blood-stasis syndrome in this study, informed by the cut-off of ≥20/51.5 (≈38.8%) reported in a validated Qi-stagnation and blood-stasis syndrome scale (sensitivity 80.35%, specificity 81.91%) (23). The diagnostic criteria for blood-stasis syndrome align with the international guidelines (24). The use of the TCM symptom score as an outcome measure in psoriasis patients with blood-stasis syndrome has been previously established (25).

**Table 2:** Traditional Chinese medicine symptom scores for blood-stasis syndrome of psoriasis vulgaris.

Symptoms	Score	
Hyperpigmented erythematous lesions	0	1
Hypertrophic and persistent skin lesions	0	1
Squamous and dry skin, dark face complexion or bluished of lip and nails	0	1
Dark menstrual blood with blood clots	0	1
Purplish dim tongue with ecchymosis	0	1
Sluggish or fine and slow pulse	0	1
Total score		

0 = absent, 1 = present.

#### 2.5.4 Mechanistic and exploratory outcomes

New-onset T2DM during follow-up will be recorded as an exploratory endpoint. Participants will be screened at Weeks 16 and 40 in accordance with the diagnostic criteria outlined in the Guideline for the Prevention and Treatment of Type 2 Diabetes Mellitus in China (2020 Edition). Furthermore, inflammatory biomarkers (serum levels of IL-1β, IL-6, TNF-α, IL-17A, and FGL1) will be analyzed at these same time points to elucidate the systemic biological pathways linking skin and metabolic health.

The recruitment, follow-up, and testing time points are presented in Table 3. To ensure objectivity and minimize assessment bias, the PASI and PGA scores will be assessed by independent, appropriately trained dermatologists who are not involved in patient management and remain entirely masked to participant group allocations. To prevent detection bias in patient-reported outcomes, the DLQI questionnaires and VAS will be administered and collected by independent clinical assessors who also remain completely blinded to group allocations. Additionally, all biochemical assays and biomarker measurements will be conducted by the hospital central laboratory personnel who are independent of the clinical research team and remain strictly blinded to participant group allocations. The conceptual framework linking the integrated intervention to the primary and secondary outcomes is presented in Figure 2.

**Table 3:** SPIRIT 2025 participant timeline.

Timepoint	Trial period													
	Enrollment		Treatment				Follow-up							Close-out
	*−7D* to *0W*	*0W*	*4W*	*8W*	*12W*	*16W*	*20W*	*24W*	*28W*	*32W*	*36W*	*40W*	*Premature termination*	*t_*x*_*
Enrollment:														
Informed consent	+													
Eligibility screen	+													
Allocation		+												
Intervention/Comparator:														
*CSG: conventional treatment*		+	+	+	+	+	+	+	+	+	+	+		
*ISG: conventional treatment*		+	+	+	+	+	+	+	+	+	+	+		
*ISG: taodan granules (TDG)*		+	+	+	+	+								
*ISG*:*fishoil*+*exercise*^a^		+	+	+	+	+	+	+	+	+	+	+		
ISG: TDG adherence assessment			+	+	+	+								
ISG: fish oil and exercise adherence assessment			+	+	+	+	+	+	+	+	+	+	+	+
Concomitant treatment review			+	+	+	+	+	+	+	+	+	+	+	+
Adverse event collection			+	+	+	+	+	+	+	+	+	+	+	+
HbA1c	+					+						+	+	
FPG	+					+						+	+	
2-h postprandial glucose	+					+						+	+	
FINS	+					+						+	+	
C-peptide	+					+						+	+	
Lipid profile 8 items^b^	+					+						+	+	
hsCRP	+					+						+	+	
IL-1β	+					+						+	+	
IL-6	+					+						+	+	
TNF-α	+					+						+	+	
IL-17A	+					+						+	+	
FGL1	+					+						+	+	
PASI score	+		+	+	+	+	+	+	+	+	+	+	+	
BSA score	+		+	+	+	+	+	+	+	+	+	+	+	
PGA score	+		+	+	+	+	+	+	+	+	+	+	+	
DLQI score	+		+	+	+	+	+	+	+	+	+	+	+	
VAS score	+		+	+	+	+	+	+	+	+	+	+	+	
TCMSS	+		+	+	+	+						+	+	
Complete blood count	+					+						+	+	
Liver function test, 3 items^c^	+					+						+	+	
Renal function test, 2 items^d^	+					+						+	+	

CSG, conventional strategy group; ISG, integrated strategy group; TDG, Taodan granules; HbA1c, glycated hemoglobin; FPG, fasting plasma glucose; FINS, fasting insulin; hsCRP, high-sensitivity C-reactive protein; IL-1β, interleukin-1 beta; IL-6, interleukin-6; TNF-α, tumor necrosis factor-alpha; IL-17A, interleukin-17A; FGL1, fibrinogen-like protein 1; PASI, psoriasis area and severity index; BSA, body surface area; PGA, physician global assessment; DLQI, dermatology life quality index; VAS, visual analog scale; TCMSS, traditional Chinese medicine syndrome score.

ISG: Fish oil + exercise^a^: Participants in the Integrated Strategy Group will continue fish oil supplementation and the prescribed exercise programme throughout both the 16-week treatment phase and the subsequent follow-up period, through Week 40.

Lipid profile 8 items^b^: TG, serum triglycerides; TC, serum total cholesterol; HDL, serum high-density lipoprotein cholesterol; LDL, serum low-density lipoprotein cholesterol; apoAI, serum apolipoprotein AI; ApoB, serum apolipoprotein B; serum lipoprotein(a); sdLDL, serum small dense low-density lipoprotein.

Liver function 3 items^c^: ALT, alanine aminotransferase, AST, aspartate aminotransferase; total bilirubin.

Kidney function 2 items^d^: creatinine, uric acid.

Source: Chan et al. (35).

© 2025 Chan A-W et al. This is an Open Access article distributed under the terms of the Creative Commons Attribution License (https://creativecommons.org/licenses/by/4.0/), which permits unrestricted use, distribution, and reproduction in any medium, provided the original work is properly cited.

**Figure 2:**
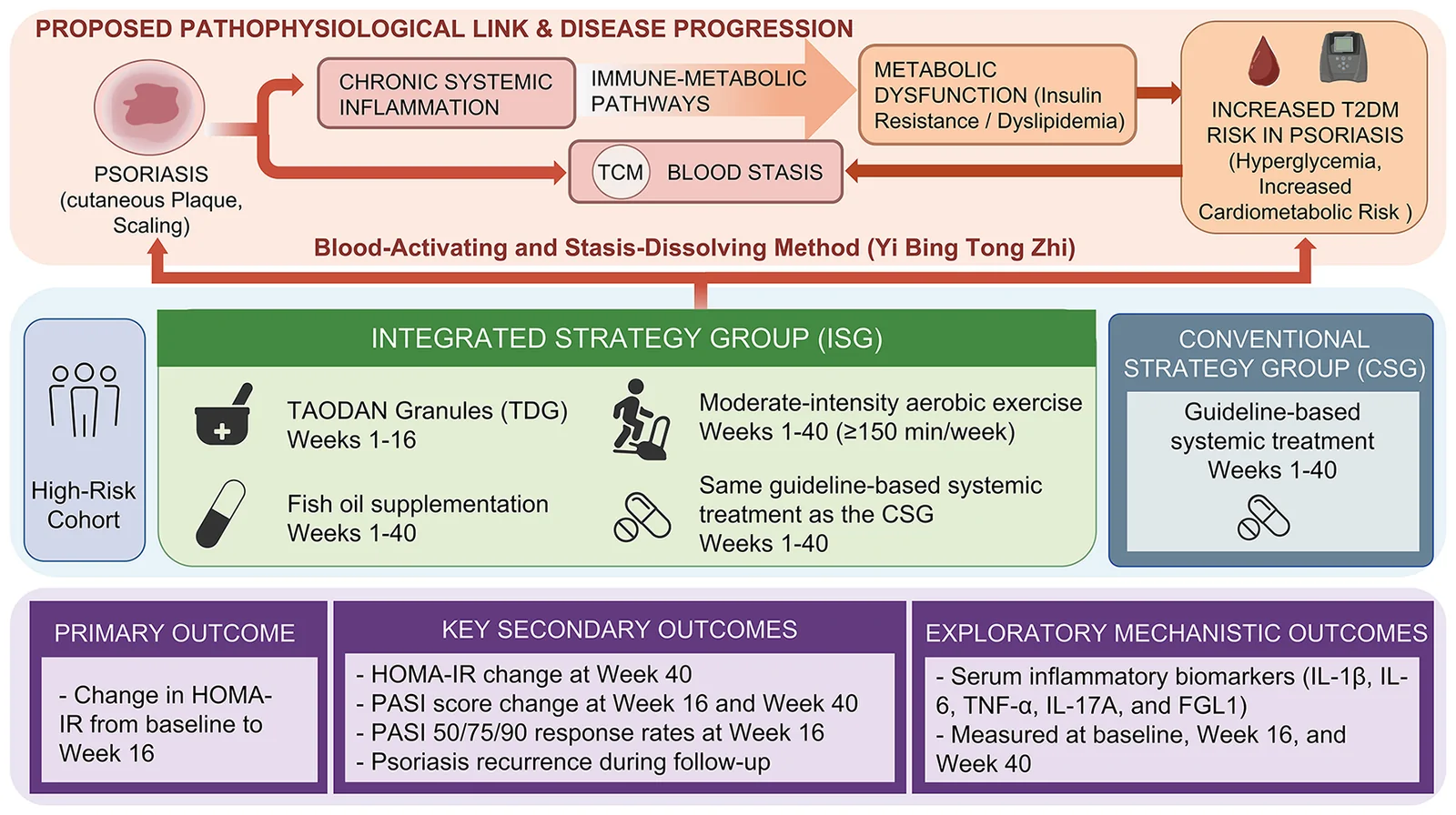
Graphical abstract. CSG, conventional strategy group; FGL1, fibrinogen-like protein 1; HOMA-IR, homeostasis model assessment for insulin resistance; IL-1β, interleukin-1β; IL-6, interleukin-6; IL-17A, interleukin-17A; ISG, integrated strategy group; PASI, psoriasis area and severity index; T2DM, type 2 diabetes mellitus; TCM, traditional Chinese medicine; TDG, Taodan granules; TNF-α, tumor necrosis factor-alpha.

### 2.6 Safety monitoring and trial suspension

Participant safety will be monitored throughout the 40-week study period. Laboratory safety indicators, including hematology, liver function (ALT, AST, TBIL), and kidney function (serum creatinine, uric acid), will be assessed at baseline and Weeks 16 and 40 to actively detect any subclinical toxicities.

An adverse event (AE) is defined as any untoward medical occurrence in a participant that does not necessarily have a causal relationship with the treatment. A Serious Adverse Event (SAE) is defined as any medical occurrence that results in death, is life-threatening, requires hospitalization, or causes persistent disability. All AEs will be recorded and graded for severity according to the Common Terminology Criteria for Adverse Events (CTCAE, version 5.0). The causality between the event and the intervention will be assessed by investigators as definitely related, probably related, possibly related, or unrelated. Any identified SAE must be reported to the Ethics Committee of Yueyang Hospital of Integrated Traditional Chinese and Western Medicine, Shanghai University of Traditional Chinese Medicine, within 24 h of awareness. The SAE reporting form is provided in Supplementary File 3.

For any AE, the investigator shall promptly assess the severity, causality, and required action. Management strategies include: (1) close observation without change to the intervention regimen (for mild, transient AEs unlikely related to the study intervention); (2) symptomatic treatment or concomitant medication (e.g., antihistamines for mild allergic reactions, topical corticosteroids for local skin irritation); (3) temporary interruption of the study intervention (e.g., withholding TDG or adjusting exercise intensity) until the AE resolves to baseline or stabilizes; or (4) permanent discontinuation of the intervention in the affected participant (e.g., for moderate-to-severe AEs that do not resolve despite symptomatic treatment, or for any SAE). All management decisions, including the rationale, duration of interruption, and subsequent follow-up, must be documented in the participant's electronic case report form. Participants who permanently discontinue the intervention will continue scheduled safety follow-up assessments per the protocol unless they withdraw consent.

The trial will be suspended or terminated early if any of the following pre-defined criteria are met: (1) any drug-related SAE occurs; (2) the investigator identifies the occurrence of other serious or life-threatening organic disorders; (3) the participant receives other experimental treatments or prohibited concomitant therapies that could interfere with safety or efficacy evaluations; (4) female participants of childbearing potential fail to adhere to the required contraceptive measures; or (5) any other significant safety risks emerge where the investigator determines that the potential risks to the subjects outweigh the clinical benefits.

Safety oversight is completely independent of the funding bodies, which have no involvement or authority in safety monitoring, trial oversight, or decisions regarding study modification, suspension, or termination. The principal investigator and study investigators are responsible for identifying, assessing, managing, and reporting adverse events and serious adverse events according to the protocol and applicable regulations. The independent monitor independently reviews protocol compliance, data quality, and safety reporting procedures, and promptly communicates any identified safety concerns to the institutional sponsor and Ethics Committee. The institutional sponsor may implement urgent protective measures or suspend study activities when required for participant safety, in consultation with the principal investigator and in accordance with applicable ethical and regulatory requirements. The Ethics Committee maintains independent authority to review safety information and may require protocol modification, temporary suspension, or termination when necessary to protect participants' rights, safety, and well-being. No Data and Safety Monitoring Board (DSMB) is required, given the low-risk nature of the interventions and the short trial duration.

### 2.7 Sample size

The sample size was estimated based on a two-sample-independent mean comparison for the primary outcome of HOMA-IR. Based on published data (26), the mean HOMA-IR in patients with severe psoriasis (PASI > 20) was 4.13 (SD 3.75). The intervention group was expected to achieve a mean HOMA-IR of 2.93, which lies between the mild and moderate psoriasis groups in the same study, representing a clinically meaningful improvement. Thus, the anticipated mean difference between groups was 1.20. To account for greater population heterogeneity in our trial, the pooled standard deviation was conservatively set at 3.20 (compared to the calculated 2.62 from the original data). With a two-sided α of 0.05 and a power of 0.80 (*Z*__α_/2_ = 1.96, *Z*_β_ = 0.84), the required sample size per group was calculated as:

$$\begin{array}{l} n=\frac{2{\left({Z_{\alpha }}_{/2}+Z_{\beta }\right)}^{2}\times {SD}^{2}}{{\left(\mu_{1}-\mu_{2}\right)}^{2}} \end{array}$$

Rounding up, 112 patients per group were needed to complete the study. Considering a 20% dropout rate common in lifestyle-intervention trials, the final recruitment target was set at 140 patients per group, yielding a total sample size of 280 patients.

### 2.8 Data management

All data generated during the clinical trial, including demographic information, clinical assessments, laboratory results, and adverse event reports, will be recorded and stored in the encrypted “Medical Road Cloud” electronic medical record system (https://sci.medroad.cn/) provided by Jiangsu Famous Medical Technology Co., Ltd. To ensure data integrity and security, we will implement a comprehensive data management strategy, as detailed below.

To maximize data accuracy and restrict unauthorized access, all research personnel will undergo standardized operational procedure training before trial initiation. Investigators can only access data from their own enrolled participants, whereas study administrators and data monitors have viewing privileges for all participants. No unauthorized personnel will have access to identifiable participant data at any time. The system incorporates predefined value ranges for all variables and automatic error notifications to maximize data accuracy. Any corrections or modifications to the electronic data after initial entry will be strictly controlled and traceably logged into the system with date, time, user identity, original value, and reason for change, constituting a complete and auditable audit trail. The audit trail will be reviewed by the study administrator at each quarterly internal monitoring visit and at the time of data lock.

Regarding protocol deviations, all events will be identified, recorded, and classified as minor or major in the electronic case report form at the time of occurrence. Major protocol deviations include, but are not limited to, patients in the CSG receiving a non-permitted primary treatment for more than four consecutive weeks. Major deviations that may affect the primary outcome will be reported to the Ethics Committee within the timeframe specified in the ethics approval. A deviation log will be maintained throughout the trial and reviewed at each monitoring visit. The handling of deviations in the statistical analysis will follow the procedures described in Section 2.9.

Upon completion of data collection and resolution of all outstanding queries, a formal data lock will be performed, followed by implementation of the archiving procedure. Prior to data lock, the study administrator and PI will conduct a final review of completeness, consistency, and audit trail integrity; once locked, no further modifications to the dataset will be permitted without documented justification and Ethics Committee notification where required. All participant data will be de-identified prior to analysis; each participant will be assigned a unique study identification number, and no directly identifiable information (name, date of birth, contact details) will appear in the analysis dataset. Data will be stored on encrypted servers with access restricted to authorized study personnel. Data will be retained for a minimum of 10 years following study completion in accordance with applicable Chinese regulations on clinical trial data archiving.

Finally, data monitoring, quality assurance, and open science principles will be strictly maintained. Data export from the electronic system will be performed by the study administrator only, in a format compatible with the planned statistical software (R version 4.4.3). An independent data monitor, who is not a member of the core research team, will verify a randomly selected 10% sample of entered data against source documents at each quarterly monitoring visit to confirm data accuracy and completeness. Internal monitoring will also be conducted quarterly, covering data completeness, protocol adherence, adverse event reporting, and audit trail integrity, with findings reported to the PI. Upon study completion, the locked, de-identified final analysis dataset, together with the complete analysis code (R scripts), will be archived in a secure institutional repository and made available to qualified researchers upon reasonable request following publication, in accordance with open science principles.

### 2.9 Statistical analysis

Statistical analyses will be performed using R (Version 4.4.3). The analysis will encompass the actual number of participants enrolled, dropout and exclusion cases, demographic and baseline characteristics, participant compliance, and both efficacy and safety profiles. Full details of the statistical analysis plan, including prespecified models, covariates, and sensitivity analyses, are provided in Supplementary File 4.

#### 2.9.1 Analysis sets and missing data

Efficacy assessments will be primarily conducted on the Full Analysis Set (FAS) following the intention-to-treat (ITT) principle. The Per-Protocol Set (PPS) will serve as a secondary set for sensitivity analysis to validate the robustness of the results. To address potential bias from the anticipated 20% dropout rate, multiple imputation will be employed to handle missing data within the FAS, and further sensitivity analyses will be conducted to evaluate the robustness of the primary findings against different missing data assumptions.

Continuous quantitative data will first be evaluated for normality using the Shapiro-Wilk test. Normally distributed variables will be expressed as mean ± standard deviation and analyzed using the independent samples *t*-test, applying the Satterthwaite correction in cases of unequal variances. Non-normally distributed data will be presented as the median with interquartile range (Q1, Q3) and compared using the Wilcoxon rank-sum test. For qualitative data, categorical variables will be reported as frequencies and percentages. Group comparisons for these categorical metrics will be performed using the Chi-square (χ^2^) test, Fisher's exact test, or the Cochran-Mantel-Haenszel chi-square (CMH χ^2^) test where appropriate.

The PPS for the Week 16 primary-outcome sensitivity analysis will comprise participants in the FAS who have a Week 16 primary-outcome assessment, have no major protocol deviation that materially affects eligibility, treatment exposure, or ascertainment of the primary outcome, and meet the prespecified adherence requirements during Weeks 1–16. For participants in the ISG, adequate adherence requires receipt of at least 80% of the scheduled TDG doses, receipt of at least 80% of the scheduled fish oil doses, and achievement of the prescribed weekly exercise target in at least 80% of the prescribed Weeks 1–16. During the Week 1–4 ramp-up period, exercise adherence will be assessed against the participant's individualized weekly target. For participants in the CSG, adherence to the assigned conventional-treatment strategy and the absence of a major treatment-related deviation will be required. Adherence or treatment changes occurring after Week 16 will not determine eligibility for the PPS analysis of the Week 16 primary outcome.

Major protocol deviations will not result in exclusion from the primary FAS analysis. The primary FAS analysis will follow a treatment-policy strategy and will include observed outcomes irrespective of treatment intensification, switching, permitted dose changes, rescue therapy, treatment discontinuation, or protocol deviations. The handling of intercurrent events and the supplementary while-on-treatment analysis are prespecified in Supplementary File 4.

#### 2.9.2 Primary and longitudinal efficacy evaluation

The sole confirmatory analysis for the primary outcome will estimate the between-group difference in the mean change in HOMA-IR from baseline to Week 16 (ISG vs. CSG). This contrast will be estimated using a linear mixed-effects model with study group, time (Weeks 0, 16, and 40), the Group × Time interaction, and the prespecified baseline covariates as fixed effects, together with a participant-level random intercept to account for repeated measurements within participants.

The primary model will be stratified by the class of conventional therapy—biologics vs. small-molecule inhibitors—in accordance with Supplementary File 4. The Group × Time interaction across Weeks 0, 16, and 40 will be treated as a secondary analysis of the longitudinal trajectory and will not supersede the prespecified Week 16 confirmatory contrast. Post-baseline exercise exposure will not be included as an adjustment covariate in the primary confirmatory model because exercise is a component of the integrated strategy and may lie on the pathway through which the strategy affects the outcome. Exercise adherence and exposure will instead be summarized by group and examined in descriptive or exploratory analyses.

Model assumptions, including the distribution and variance of residuals, will be assessed using appropriate diagnostic plots. If material violations are identified, prespecified transformations or robust standard errors will be considered and reported transparently. Missing primary-outcome data will be handled using multiple imputation under the missing-at-random assumption, with complete-case analysis conducted as a sensitivity analysis. Full details of the analysis sets, covariates, intercurrent-event strategies, and sensitivity analyses are provided in Supplementary File 4.

#### 2.9.3 Propensity score matching as sensitivity analysis

To further mitigate selection bias arising from the non-randomized, preference-based allocation, propensity score matching will be performed as a sensitivity analysis. Within each conventional-therapy stratum (biologics vs. small-molecule inhibitors), 1:1 nearest-neighbor matching without replacement will be conducted using a fixed caliper of 0.20 standard deviations of the logit of the estimated propensity score. The propensity-score model will include the following prespecified baseline covariates: age, sex, BMI, baseline PASI score, disease duration, baseline HOMA-IR, baseline fasting plasma glucose, baseline lipid parameters (TC, TG, HDL-C, and LDL-C), prior psoriasis treatment history (topical therapy only vs. systemic therapy/biologics), and the T2DM risk score derived from the previously validated multifactorial model. No covariate will be selected or removed on the basis of statistical significance or the observed outcomes.

Covariate balance will be considered acceptable only when the absolute standardized mean difference is < 0.10 for every prespecified covariate. If this criterion is not achieved, the caliper will not be modified after inspection of the data. The remaining imbalance and the number of unmatched participants will be reported, and the PSM result will be labeled as not adequately balanced and interpreted as exploratory. Unmatched participants will be excluded only from the PSM sensitivity analysis and will remain in the primary FAS analysis.

Outcomes in the matched sample will be analyzed using the prespecified linear mixed-effects model with random intercepts at the matched-pair and participant levels to account for within-pair dependence and repeated measurements within participants. Variance inflation factors will be reported as model diagnostics. Continuous covariates may be centered to improve numerical stability, but no prespecified covariate will be removed solely because of its variance inflation factor. No inverse-probability-weighted analysis is planned.

#### 2.9.4 Hypothesis testing

All hypothesis tests will be two-sided. Results will be reported with test statistics and their corresponding *P*-values. Statistical significance is defined as *P* ≤ 0.05, and *P* ≤ 0.01 will be considered highly statistically significant.

## 3 Discussion

The co-management of psoriasis and diabetes remains a clinical challenge. Current management strategies often rely on fragmented therapeutic approaches, such as biologics or topical treatments for skin lesions and glucose-lowering agents for hyperglycaemia, but lack an integrated intervention that addresses their shared pathophysiological basis. Previous studies indicated that omega-3 polyunsaturated fatty acids in fish oil supplements may ameliorate disease activity through their anti-inflammatory and lipid-modulating properties. A meta-analysis of randomized controlled trials reported that omega-3 supplementation significantly reduced psoriasis severity, including PASI score, erythema, and scaling (27). Our systematic review also found that combining fish oil with conventional therapy reduced psoriasis severity and modulated related comorbidities (18). Similarly, our meta-analysis found that moderate- to high-intensity exercise was associated with improvements in psoriatic lesions and a lower risk of prevalent psoriasis. A recent meta-analysis has further demonstrated that post-exercise PASI score decreased significantly in psoriasis patients, which supports physical activity intervention as a therapeutic option (28). A 20-week physical activity intervention significantly reduced the PASI score, with 50% of participants achieving PASI 50 (29). Nevertheless, the combination of these complementary non-pharmacological interventions with pharmaceutical regimens guided by TCM theory has not been adequately validated in prospective studies. Therefore, this study aims to address this gap by evaluating a practical and evidence-based intervention protocol that integrates TCM with conventional medicine for the co-management of psoriasis and diabetes.

This study was designed based on the shared pathophysiological mechanisms underlying psoriasis and diabetes, particularly the dysregulated “immune–metabolic–inflammatory” pathways common to both conditions. First, TDG may act through several therapeutic mechanisms. Integrated mechanistic studies revealed that TDG upregulates the Gly-Ser-Thr metabolic axis while downregulating key pro-inflammatory pathways such as IL-17, TNF-α, and chemokine signaling, leading to decreased levels of cytokines including IL-6 and TNF-α (30). Furthermore, transcriptomic analyses identified suppression of the Wnt pathway as a core mechanism, whereby TDG downregulates upstream regulators (EZH2, CTNNB1, and WDR5), upregulates TP63, and inhibits the downstream effectors RAC2 and WNT5A, which are critically involved in keratinocyte hyperproliferation and migration (31). Second, nutritional supplementation with omega-3 polyunsaturated fatty acids (abundant in fish oil) improves clinical outcomes in psoriasis, including reduced PASI scores and relief from itching (32). Third, as a foundational lifestyle intervention, regular physical activity helps alleviate psychological distress and systemic immune activation in psoriasis while also improving metabolic parameters such as obesity and cardiovascular risk, thereby indirectly mitigating disease progression (19). Together, these mechanisms, including pathway-specific inhibition, anti-inflammatory activity, and metabolic regulation, may alleviate psoriatic dermatitis (33).

This study integrates a previously developed diagnostic prediction model with a prospective interventional protocol. Building on our earlier model for identifying psoriasis patients at high risk of T2DM, the present study will evaluate its utility in a real-world clinical setting while testing whether model-guided intervention can improve outcomes in the identified high-risk cohort (15). The operational validation comprises two components: evaluating the model's calibration within the CSG and assessing outcomes among model-identified high-risk individuals who receive the tailored intervention. Our objective is to evaluate a closed-loop management approach that links risk prediction to the delivery of a comprehensive intervention for identified high-risk individuals. This approach may support stratified management and the prevention of comorbidities in patients with psoriasis.

Several limitations of this study should be acknowledged. First, because the intervention comprises multiple components, participants and treating clinicians cannot be blinded, which may introduce performance and expectation bias. To mitigate these risks, the change in HOMA-IR, an objective metabolic measure, was selected as the primary outcome. Independent clinicians who are not involved in patient management and are blinded to group allocation will perform the PASI assessments and administer the DLQI. All laboratory analyses will be conducted by personnel from the hospital central laboratory, who will also remain independent of the core research team and blinded to group allocation. These measures are intended to reduce detection bias and support the objectivity of the clinical, biochemical, and quality-of-life data.

Second, as a prospective patient preference-based cohort study, the non-randomized design may inherently lead to baseline imbalances and residual confounding. While we employ LMM/DID models with a prespecified covariate set to statistically adjust for these factors and additionally perform PSM as a sensitivity analysis (Section 2.9), the possibility of residual confounding cannot be entirely ruled out. Relatedly, although conventional treatment in the CSG has been restricted to two defined classes (biologics and small-molecule inhibitors) and is stratified in the primary analysis, some residual heterogeneity in specific agents and dosing within each class is unavoidable under a pragmatic design. Furthermore, the integrated nature of this strategy makes it challenging to isolate the independent contribution of each component (blood-activating and stasis-dissolving therapies, fish oil, or exercise) at this stage.

Third, the use of self-purchased fish oil and real-world exercise introduces residual exposure variability. We have specified minimum EPA/DHA (eicosapentaenoic acid/docosahexaenoic acid) content, formulation, purity, and storage requirements for fish oil, and a wearable-monitored adherence threshold (≥80%) for exercise, with verification at each visit (Section 2.3); nonetheless, full standardization of these components cannot be guaranteed in a pragmatic design.

Fourth, the 40-week follow-up period is insufficient to record long-term clinical events such as T2DM incidence. However, it provides a robust window to evaluate critical surrogate markers and short-to-medium-term metabolic shifts. Because fish oil supplementation and the prescribed exercise programme continue in the ISG throughout Weeks 16–40 after TDG discontinuation, the Week 40 assessment represents outcomes under continued lifestyle intervention after TDG discontinuation. It cannot isolate a TDG-specific residual effect and should not be interpreted as a complete off-treatment assessment.

Finally, given the number of secondary and exploratory endpoints, no formal multiplicity adjustment is applied; these are interpreted as hypothesis-generating, with confirmatory inference restricted to the primary outcome. The sample size was calculated from external published data with assumed effect size and dropout rate, and actual attrition may differ, which will be addressed through the prespecified missing-data sensitivity analyses (Section 2.9.1). Full ELISA assay specifications for the inflammatory biomarker panel will be finalized and documented before trial commencement (Section 2.4); these biomarkers are accordingly treated as exploratory outcomes.

We will maintain data integrity through rigorous adherence monitoring and standardized follow-up protocols. The reporting of this study follows the SPIRIT 2025 Statement, the SPIRIT extension for Chinese herbal formula trials (SPIRIT-CHF 2025), and the TIDieR guideline to improve the completeness and transparency of protocol reporting (34).

## Study oversight

Yueyang Hospital of Integrated Traditional Chinese and Western Medicine, Shanghai University of Traditional Chinese Medicine, serves as the institutional sponsor of this study and provides the research platform and overall institutional oversight. The principal investigator and study investigators are responsible for the scientific conduct of the study, protocol implementation, participant management and protection, assessment and reporting of adverse events, maintenance of data integrity, and compliance with the approved study protocol. The independent monitor, who is separate from the core research team, independently reviews protocol compliance, data quality, and safety reporting, and provides monitoring reports to the appropriate oversight bodies. The Ethics Committee maintains independent ethical oversight throughout the study, reviews safety information, protocol deviations, and other ethical issues, and has the authority to require protocol modifications, temporary suspension, or termination of the study when necessary to protect participants' rights, safety, and well-being. Decisions regarding modification, temporary suspension, or early termination of the study for safety, ethical, or regulatory reasons will be made by the institutional sponsor in consultation with the principal investigator and in accordance with the requirements of the Ethics Committee and applicable regulations. The funding bodies have no authority or involvement in such decisions.

## Data Availability

The original contributions presented in the study are included in the article/Supplementary material, further inquiries can be directed to the corresponding authors.
