# Supplementary material for: Protocol for a cohort study of comprehensive intervention of psoriasis and diabetes comorbidity prevention and control using traditional Chinese medicine of blood-activating and stasis-dissolving method
Source: Front Med (Lausanne). 2026 Oct 14;13:1831495. doi: 10.3389/fmed.2026.1831495 (PMC13717394; doi:10.3389/fmed.2026.1831495)
Supplement: Supplementary file 1 [file Supplementary_File_1.docx]

**Consent Form**

Study title: Protocol for a Cohort Study of Comprehensive Intervention of Psoriasis and Diabetes Comorbidity Prevention and Control Using Traditional Chinese Medicine of Blood-Activating and Stasis-Dissolving Method

Research institution: Yueyang Hospital of Integrated Traditional Chinese and Western Medicine, Shanghai University of Traditional Chinese Medicine

Principal investigator: Xin Li

Address: No. 110 Ganhe Road, Hongkou District, Shanghai

Dear Participant,

You have been diagnosed with psoriasis vulgaris (plaque psoriasis).

We invite you to consider taking part in this study on the prevention and management of type 2 diabetes mellitus (T2DM) in people with psoriasis.

Before you decide, please read this information carefully. It explains why the study is being done, what will happen if you take part, how long the study will last, and the possible benefits, risks, and inconveniences. Please ask the study doctor about anything you do not understand. You may also discuss the study with your family or friends before making your decision.

**Ⅰ. Why is this study being conducted?**

**1. Background**

Psoriasis is a long-term inflammatory condition that affects the skin and may also be associated with metabolic conditions such as obesity and T2DM. People with more extensive or severe psoriasis may have a higher risk of developing T2DM. Traditional Chinese medicine (TCM), fish oil supplementation, and regular exercise have each been studied as possible ways to improve psoriasis-related or metabolic outcomes. However, the combined strategy used in this study has not yet been evaluated in a prospective study.

**2. Purpose of the study**

This study will evaluate whether an integrated strategy comprising Taodan granules (TDG), fish oil supplementation, and moderate-intensity aerobic exercise, in addition to permitted conventional psoriasis treatment, can improve insulin resistance and psoriasis outcomes in people with psoriasis who are at high risk of T2DM. The study will be conducted at Yueyang Hospital of Integrated Traditional Chinese and Western Medicine. We plan to enrol 280 participants, with a target of 140 participants in each group. The study has been reviewed and approved by the hospital ethics committee (approval no. 2024-034).

**II. Who can and cannot take part?**

**1. You may be eligible if all of the following apply:**

(1) You meet the diagnostic criteria for psoriasis vulgaris with TCM blood-stasis syndrome.

(2) Your psoriasis may be newly diagnosed or previously diagnosed. There is no restriction on your previous psoriasis treatment.

(3) The study risk model identifies you as being at high risk of T2DM, and you have not previously been diagnosed with diabetes.

(4) You are 18-80 years of age.

(5) You are willing to take part and sign this informed consent form.

**2. You cannot take part if any of the following apply:**

(1) You have been diagnosed with T2DM.

(2) You have another active or progressive skin condition that may interfere with assessment of your psoriasis.

(3) You have a severe or uncontrolled local or systemic infection, whether acute or chronic.

(4) You have a significant systemic illness or a clinically important laboratory abnormality, including any of the following:

haemoglobin, platelet count, white blood cell count, or absolute neutrophil count below the lower limit of normal;

• alanine aminotransferase or aspartate aminotransferase more than three times the upper limit of normal;

• total bilirubin more than 1.5 times the upper limit of normal;

• serum creatinine more than 1.5 times the upper limit of normal;

•another clinically important laboratory abnormality that, in the investigator’s judgement, may affect your safety or the study assessments.

(5) You have a history of malignant cancer, primary or secondary immune deficiency, or a hypersensitivity reaction that makes participation unsuitable.

(6) You had major surgery within the 8 weeks before the study or are scheduled to have major surgery during the study.

(7) You are pregnant or breastfeeding.

(8) You have a history of alcohol or drug abuse.

(9) You have a personal history of serious mental illness.

(10) The investigator considers that another medical or practical reason makes participation unsuitable for you.

**III. What will happen if I take part?**

**1. Screening**

The screening period will last approximately 1 week. The study team will ask about your medical history and current medicines, examine you, record your vital signs, and arrange the required laboratory tests. These tests include blood counts, liver and kidney function, glucose metabolism, and lipid measurements. If you could become pregnant, a pregnancy test will also be required.

**2. Assignment to a study group**

The study doctor will explain both treatment strategies to you. If you are eligible, you will enter either the conventional strategy group (CSG) or the integrated strategy group (ISG). You will not be assigned to a group by chance. Instead, you and your doctor will decide together which treatment strategy is most appropriate for you.

**3. Conventional strategy group (CSG)**

If you enter the CSG, your doctor will choose a guideline-based systemic treatment according to your condition. The permitted primary treatments are biologics (such as adalimumab, secukinumab, or ustekinumab) or small-molecule inhibitors (such as apremilast or deucravacitinib). Topical treatment alone, phototherapy, methotrexate, cyclosporine, and other conventional systemic medicines will not be used as your primary study treatment. You will not receive TDG, study-directed fish oil supplementation, or the structured exercise programme.

Your treatment response will be assessed at Week 16. If you have not achieved at least a 50% improvement in your Psoriasis Area and Severity Index score (PASI 50), your doctor may increase or otherwise intensify treatment within the same permitted treatment class. The intensified regimen will be maintained from Week 16 until the Week 28 reassessment, unless a change is required for safety or another clinically compelling reason. If you still have not achieved PASI 50 at Week 28, your doctor may prescribe a study-permitted topical rescue treatment. Oral antihistamines may be used at any time if you have severe itching.

**4. Integrated strategy group (ISG)**

If you enter the ISG, you will receive the same guideline-based systemic treatment as the CSG, together with the following additional interventions:

(1) Taodan granules (TDG): the daily formula contains 10 g Semen Persicae, 30 g Radix Salviae Miltiorrhizae, 10 g Rhizoma Ligustici Chuanxiong, 30 g Rhizoma Curcumae, 15 g Radix Astragali seu Hedysari, 15 g Radix Angelicae Sinensis, 15 g Radix Cyathulae, 30 g Smilacis China, and 10 g Radix Glycyrrhizae Preparata. TDG is supplied every 4 weeks. Each daily dose is divided into two compartments. Take one compartment after breakfast and one after the evening meal. Dissolve one compartment in approximately 50 mL of warm water, stir until mostly dissolved, add approximately 100 mL of hot water, and drink the solution. You will take TDG during Weeks 1-16 only.

(2) Fish oil: you will purchase the fish oil yourself and take 1 g twice daily, with the morning and evening meals, from baseline through Week 40. The study team will explain the product requirements. Please keep the original packaging and remaining capsules and bring them to study visits.

(3) Exercise: you will follow an individualised, moderate-intensity aerobic exercise programme. During Weeks 1-4, you will gradually work towards the target. From Week 5 through Week 40, the target is at least 150 minutes of moderate-intensity aerobic exercise each week. Suitable activities may include brisk walking, cycling, low-impact aerobics, or stepping exercises. If you have psoriatic arthritis or another physical limitation, the study doctor may recommend a lower-impact activity. You will record exercise in a diary and use the wearable device provided by the study team.

**5. Study duration and visits**

Your participation will include a 16-week treatment period followed by a 24-week follow-up period, for a total of 40 weeks. Study visits are scheduled at baseline and approximately every 4 weeks: Weeks 4, 8, 12, 16, 20, 24, 28, 32, 36, and 40. At these visits, the study team will ask about your health and medicines, assess your psoriasis, review any side effects, and check your adherence to the assigned treatment.

At Weeks 16 and 40, you will have clinical assessments and laboratory tests. If your treatment is intensified at Week 16, the intensified regimen will be maintained from Week 16 until the Week 28 reassessment, unless a change is required for safety or another clinically compelling reason. Its effect will be formally reassessed at Week 28. Study-permitted topical rescue treatment may be offered at Week 28 if you still have not achieved PASI 50.

If you are in the ISG, you will stop TDG after Week 16 but will continue fish oil and the prescribed exercise programme through Week 40. If you are in the CSG, you will not begin study-directed fish oil supplementation or the structured exercise programme during follow-up. Any necessary changes to your psoriasis treatment will be decided by your doctor and recorded.

**6. What do I need to do?**

Please attend the scheduled visits and tell the study team honestly about changes in your health, symptoms, medicines, and any problems you experience. Before scheduled fasting blood tests, you must fast for at least 8 hours; small amounts of water are allowed.

Follow the study doctor’s instructions. Bring all unused study medicine, TDG packaging, fish oil packaging and remaining capsules, your exercise diary, and the wearable device records requested by the study team. Tell the study doctor before starting another treatment whenever possible. Medically necessary treatment will never be withheld.

If you could become pregnant, use appropriate contraception as instructed by the study doctor. Tell the study team immediately if you think you may be pregnant.

**IV. What are the possible benefits?**

You may or may not receive a direct medical benefit from taking part. Your condition will be monitored regularly, and the information collected may help researchers understand whether this integrated strategy is useful for people with psoriasis who are at high risk of T2DM. The results may benefit patients with similar conditions in the future.

**V. What are the possible risks and inconveniences?**

TDG may cause gastrointestinal symptoms such as diarrhoea, vomiting, or stomach pain. Other possible problems include allergic reactions, skin irritation from topical treatment, discomfort related to exercise, and temporary pain, bruising, dizziness, or bleeding from blood collection. Other expected or unexpected medical problems may also occur.

Tell the study doctor promptly about any discomfort, new symptom, change in your condition, or other medical problem, whether or not you think it is related to the study. The study team will assess the problem and arrange appropriate care. Study visits, blood collection, examinations, diaries, returning packaging, and use of a wearable device may take time and cause inconvenience. The research institution will make every reasonable effort to prevent and treat study-related injury. If an expert committee determines that an injury is related to this study, the research team will provide corresponding financial compensation for the study-related injury.

**VI. What costs and payments are involved?**

The research team will pay for study-required examinations, including the required blood, urine, liver-function, and kidney-function tests. If you enter the ISG, the research team will provide TDG at no cost and will lend you the wearable device. You must purchase the fish oil yourself. Costs for treatment or tests required for medical conditions unrelated to the study will not be paid by the research team. If you complete the Week 16 visit as scheduled, you will receive RMB 200 for transportation expenses. If you continue and complete the Week 40 visit, you will receive an additional RMB 100 for transportation expenses.

**VII. How will my privacy be protected?**

Your study information will be stored in a secure, encrypted electronic system. You will be identified in the research dataset by a study number rather than by your name. Authorised investigators, study monitors, sponsor representatives, and the ethics committee may review your medical records to check that the study is being conducted correctly. They must protect your confidentiality. Reports or publications from this study will not identify you. Study data will be retained for at least 10 years after the study is completed, in accordance with applicable Chinese regulations.

Blood samples collected for this study will be stored securely and used only for the tests described in this consent form. To reduce variation between ELISA assay batches, samples may be stored until they can be analysed in the planned batches. After all planned analyses, including any prespecified quality-control repeat testing, have been completed, all remaining biological samples will be destroyed in accordance with hospital regulations. They will not be retained or used for future research.

**VIII. How can I obtain more information?**

You may ask questions about the study at any time. The study doctor will provide contact details so that you can ask questions or report a problem.

The study team will tell you about any important new information that may affect your willingness to continue taking part.

**IX. Is participation voluntary, and can I withdraw?**

Taking part is entirely voluntary. You may refuse to participate or withdraw your consent at any time. Your decision will not affect your relationship with your doctor, your usual medical care, or any benefits to which you are entitled. If you stop the study intervention, the study team may ask you to complete safety follow-up assessments, but you may also withdraw consent for further participation.

**X. What should I do now?**

Take as much time as you need to decide. You may discuss the study with your family, friends, or another doctor.

Ask the study doctor about anything you do not understand. Do not sign the consent form until your questions have been answered and you feel that you understand what participation involves. If you decide to participate, you and the investigator will sign and date the consent form, and you will receive a signed copy.

**XI. Ethics committee contact**

This study has been approved by the hospital ethics committee. If you have any question about your rights as a participant or wish to make a complaint, you may contact the hospital ethics committee (Telephone Number: 021-65161782*8122, Dr. Yin). Please keep this information sheet for your records.

**Informed Consent Signature Page**

Study title: Protocol for a Cohort Study on Comprehensive Intervention of Psoriasis and Diabetes Comorbidity Prevention and Control Using Traditional Chinese Medicine of Blood-Activating and Stasis-Dissolving Method

Principal investigator: Xin Li

Ethics approval no.: 2024-034

**Participant consent statement**

I have read the participant information sheet, or it has been read to me. I have had enough time to consider participation and have had the opportunity to discuss the study and ask questions. My questions have been answered to my satisfaction.

I understand the purpose of the study, what I will be asked to do, the possible benefits, risks, and inconveniences, and how my information and samples will be handled. I understand that taking part is voluntary.

I understand that I may ask for more information or withdraw my consent at any time without penalty and without affecting my medical care or rights. If I stop the study intervention, I may be asked to tell the study doctor about changes in my condition and to complete a safety assessment, but I may refuse further participation.

I understand that authorised investigators, study monitors, sponsor representatives, and the ethics committee may review my study and medical records while protecting my confidentiality.

I understand that my biological samples will be used only for the tests described in this study. After all planned analyses, including any prespecified quality-control repeat testing, have been completed, any remaining samples will be destroyed in accordance with hospital regulations. My samples will not be retained or used for future research.

I will receive a signed and dated copy of this consent form. By signing below, I freely agree to take part in this study.

Additional blood collection: At baseline, Week 16, and Week 40, one additional tube of blood will be collected to measure inflammatory biomarkers related to psoriasis and T2DM.

Signature of the Study Participant (handwritten):

Date:

Telephone Number of the Study Participant:

Signature of the Legally Authorized Representative (if applicable, handwritten):

Date:

Telephone Number of the Legally Authorized Representative:

Investigator statement: I confirm that I have explained the study to the participant, including the purpose, procedures, rights, possible benefits, risks, and alternatives. I have answered the participant’s questions and provided a signed copy of this consent form.

Signature of the Investigator (person obtaining informed consent from the study participant, handwritten):

Date:

Telephone Number of the Investigator:
