# Supplementary material for: Protocol for a cohort study of comprehensive intervention of psoriasis and diabetes comorbidity prevention and control using traditional Chinese medicine of blood-activating and stasis-dissolving method
Source: Front Med (Lausanne). 2026 Oct 14;13:1831495. doi: 10.3389/fmed.2026.1831495 (PMC13717394; doi:10.3389/fmed.2026.1831495)
Supplement: Supplementary file 2 [file Supplementary_File_2.docx]

**Supplementary File 2. Participant Compliance Management Manual**

**Dear Participant**

Part 1 explains the study visits and treatment information that apply to everyone in the study. If you are in the Integrated Strategy Group, you will also take Taodan granules and fish oil and follow an exercise plan. You will take Taodan granules during Weeks 1–16. Fish oil and exercise will begin at the start of the study and continue through Week 40. More details are provided in Parts 2–5.

This handbook explains exactly what you need to do. Please read it carefully and keep it with you throughout the study. If you have any questions or feel unwell at any time, please contact the research team immediately.

If you are in the Conventional Strategy Group, you will not receive Taodan granules, fish oil, or the study exercise plan. Please read Part 1 carefully because the information about study visits, treatment changes, and additional treatment also applies to you.

**Part 1: Information for Everyone in the Study**

**1.1 Attending Your Study Visits**

Please attend your scheduled visits at Weeks 4, 8, 12, 16, 20, 24, 28, 32, 36, and 40. If you cannot attend, please contact the research coordinator as soon as possible so that another suitable time can be arranged.

**1.2 Tell Us About Any Changes to Your Psoriasis Treatment**

At every visit, please tell the research team about any psoriasis treatment you have used since your previous visit. This includes treatment prescribed at our hospital, treatment prescribed elsewhere, and medicines you bought yourself. Please report the following information whenever possible:

- The name of the treatment or medicine
- When you started or stopped it
- Any dose change or change in frequency
- Whether it was prescribed by a doctor or taken on your own
- The reason for the change

If possible, please bring medicine boxes, prescriptions, discharge records, or photos of product labels to your appointment. The research team will record this information in your study record so that your treatment and study results can be reviewed accurately.

**1.3 What Happens If Psoriasis Is Not Relieved Sufficiently**

Your study doctor will choose a conventional psoriasis treatment for you: either a biologic medicine or a small-molecule medicine. Your psoriasis will be assessed regularly using the Psoriasis Area and Severity Index (PASI), a score that measures the extent and severity of your skin condition. At your Week 16 visit, the study doctor will compare your PASI score with your score at the start of the study. If your PASI score has not decreased by at least 50%, the doctor may adjust your psoriasis treatment by switching you to another permitted medicine within the same therapeutic category, according to your clinical condition and applicable treatment guidelines. The intensified regimen will be maintained from Week 16 until the Week 28 reassessment, unless a change is required for safety or another clinically compelling reason.

The effect of the adjusted treatment will be assessed at Week 28. If the PASI score still has not decreased by at least 50% from your starting score, your doctor may add an approved treatment applied to the skin, such as a strong corticosteroid cream or ointment, calcipotriol or another topical vitamin D treatment, 3% salicylic acid, or a topical retinoid such as tazarotene. These topical treatments may be continued through Week 40 if needed. Topical retinoids applied to the skin are permitted, whereas systemic retinoids taken by mouth, such as acitretin, are not permitted under the study treatment plan.

- **Please do not start, stop, change, or increase any psoriasis treatment on your own.** Contact your study doctor or the research coordinator first, unless you need urgent medical care.
- **Please do not begin other psoriasis treatments on your own:** phototherapy, oral corticosteroids, oral retinoids (such as acitretin), cyclosporine, methotrexate, or another psoriasis medicine that is not part of your study treatment, unless your doctor considers it medically necessary. Please contact the study team before starting any new psoriasis treatment whenever possible. (Topical retinoids such as tazarotene, applied to the skin as described above, are different from oral retinoids and are permitted as part of your study treatment if needed.)

Please tell the study team if you use any treatment that is not included in the study plan, including the name of the treatment and how long you used it. This information is important for your safety and for the accurate assessment of the study results.

**1.4 If You Are in the Conventional Strategy Group**

If you are in the Conventional Strategy Group, you will continue the psoriasis treatment prescribed by your study doctor. Your treatment may be adjusted, or additional skin treatment may be added if needed, as explained in Section 1.3. You will not receive Taodan granules, fish oil, or the study exercise plan. Please continue attending all visits and reporting any treatment changes through Week 40.

**Part 2: How to Take Taodan Granules (TDG)**

**2.1 What Are Taodan Granules?**

Taodan granules are a Chinese herbal formula made from several individual herbs prepared as concentrated granules. In this study, Taodan granules are being used according to the traditional Chinese medicine principle of activating blood circulation and resolving blood stasis. The study will evaluate their possible effects on psoriasis and related metabolic health. The granules are produced by Sichuan Neo-green Pharmaceutical Technology Development Co., Ltd. Quality testing is performed before the study medicines are provided to participants.

**2.2 How to Take TDG**

- **Dose frequency:** Take TDG twice daily — once in the morning and once in the evening.
- **How to prepare each dose:** Pour one compartment (half the daily dose) into approximately 50 mL of warm water. Stir until mostly dissolved, then add another 100 mL of hot water and stir again. Drink the solution straight away.
- **Timing:** Take TDG after meals (after breakfast in the morning, after dinner in the evening).
- **Packaging:** Each box contains seven single-dose compartments. Because you take two doses each day, one box lasts three and a half days. At each 4-week visit, you will normally receive eight boxes, providing 28 days of treatment.

**2.3 Collecting and Storing Your Medication**

- Medications will be dispensed every 4 weeks at your scheduled clinic visit.
- **Important:** Please bring back all empty boxes, any unused compartments, and any unopened boxes at each 4-week visit. The research coordinator will check them before giving you the next supply.
- Store TDG at room temperature, away from direct sunlight, moisture, and heat. Do not open multiple boxes at once.
- Each box is labelled with a batch number and expiry date. Please check the expiry date before use and do not use expired products.

**2.4 What to Do If You Miss a Dose**

- If you forget one dose, skip it and take the next dose as scheduled. Do not double up the next dose to make up for a missed one.
- Please record any missed doses honestly in the weekly diary table at the end of this handbook. Missing an occasional dose will not disqualify you from the study, and accurate recording helps the research team support you.

**2.5 When to Contact the Research Team**

Please contact the research team if you develop stomach pain, nausea, vomiting, diarrhoea, a skin rash, itching, or another unusual symptom after taking Taodan granules. The study doctor will advise you whether to continue, pause, or stop the medicine. If the symptoms are severe, seek medical care promptly.

**Part 3: How to Choose and Take Fish Oil Supplements**

**3.1 Why Take Fish Oil?**

Fish oil contains omega-3 fatty acids called EPA and DHA. In this study, fish oil is included because it may help support skin and metabolic health. Because fish oil products vary widely in quality and content, please follow the guidance below when choosing a product. The research team will check your product at every clinic visit.

**3.2 Dose**

- **Take a total of 1 g of fish oil in the morning and 1 g in the evening**, with meals, for a total of 2 g each day. The number of capsules needed depends on the amount contained in each capsule. The research team will confirm the correct number of capsules for the product you purchase.

**3.3 How to Choose a Suitable Product**

Before buying fish oil, please show the product label or product information to the research team. The team will help you check whether the product is suitable.

The product should provide at least 180 mg of EPA and 120 mg of DHA in each 1 g of fish oil. Please choose a product from a legally registered manufacturer with a clearly labelled batch number and expiry date. Do not change to another fish oil product without informing the research team.

**3.4 Storage Instructions**

- Store fish oil capsules in a cool, dark location away from heat and direct sunlight (recommended storage temperature: ≤ 25°C).
- Always close the bottle tightly after each use to prevent oxidation.

**3.5 What to Bring to Each Clinic Visit**

At every 4-weekly clinic visit, please bring:

- The original packaging of the fish oil product you are currently using (bottle or box), including empty bottles/blister packs and any remaining unused capsules.

The research coordinator will record the product name, manufacturer, declared EPA/DHA content, batch number, and expiry date, and will count the remaining capsules to calculate your adherence. If the product is not suitable for the study, the research team will explain why and help you choose another suitable product.

**3.6 Side Effects to Watch For**

Fish oil is generally well tolerated. However, please inform the research team if you notice any of the following: persistent fishy aftertaste, stomach discomfort (nausea, bloating, or loose stools), skin rash or itching, or any increase in bleeding tendency (such as unusual bruising, nosebleeds, or prolonged bleeding from minor cuts). Any such events will be recorded and managed according to the study safety procedures. Seek medical care promptly if you have heavy or unexplained bleeding.

**Part 4: How to Exercise Safely and Effectively**

**4.1 Why Is Exercise Part of the Study?**

Regular moderate-intensity exercise may support skin and metabolic health. As part of this study, you will follow an exercise plan prepared with the research team.

**4.2 Your Weekly Exercise Target**

- **Aim for at least 150 minutes of moderate-intensity aerobic exercise each week.**
- **Target heart rate during exercise:** approximately 60%–70% of your estimated maximum heart rate.
- **How to estimate your maximum heart rate:** subtract your age from 220. For example, if you are 50 years old, the estimated maximum is 170 beats per minute (bpm), and the target range is approximately 102–119 bpm. The research team will help you calculate your personal range.

**4.3 Structure of Each Exercise Session**

For safety, each exercise session should include the following three parts:

- **Warm-up (5 minutes):** Start with gentle stretching and slow walking to prepare your body for exercise.
- **Core aerobic exercise:** Perform your chosen aerobic activity at a pace that keeps your heart rate within the 60–70% target zone. Your ACVOR wearable device will display your real-time heart rate to help you stay on track.
- **Cool-down (5 minutes):** Finish with gentle stretching and slow walking to help your heart rate and breathing return to normal.

Note: When recording your weekly exercise time, record the time spent in the main aerobic part of each session. Do not include the warm-up or cool-down periods.

**4.4 Recommended Types of Exercise**

- Brisk walking (approximately 3.5–5.0 km/h), slow-pace cycling (< 16 km/h), low-impact aerobics, or stepping exercises.
- **If brisk walking or cycling is difficult because of joint pain or another health problem,** the study doctor may recommend a lower-impact activity, such as Baduanjin (Eight-Section Brocade), Wuqinxi (Five Animals Exercise), Yijinjing (Muscle-Tendon Changing Classic), or Tai Chi. The research team will help you choose a low-impact activity that is suitable for your health and physical ability.
- Your responsible physician will assess the most appropriate exercise type for you at the start of the study and will review your exercise plan at each clinic visit.

**4.5 How to Build Up During the First 4 Weeks (Ramp-Up Phase)**

We understand that everyone has a different starting level of fitness. During Weeks 1–4, you do not need to reach 150 minutes immediately. Instead, gradually increase your exercise duration week by week at a pace that feels comfortable, while aiming to keep your heart rate within the target zone. From Week 5 onward, please aim to complete at least 150 minutes each week. After the Week 16 visit, continue the same weekly target as explained in Part 5.

**4.6 Using Your ACVOR Wearable Device**

- The research team will provide you with an ACVOR wearable device free of charge at your first visit. Please wear it during all exercise sessions throughout the study.
- The device records your exercise time and heart rate. The research team will show you how to view or upload the records using the ACVOR app, where applicable.
- **How an exercise session is counted:** An exercise session will count toward your weekly total when your heart rate remains within your target range for at least 70% of the main exercise period. For example, during a 30-minute main exercise period, your heart rate should remain in the target range for at least 21 minutes.
- At each 4-weekly clinic visit, the research coordinator will review your ACVOR app data together with your paper diary. If there are any differences between the two records, the coordinator will discuss these with you.
- If your records show that you are having difficulty meeting the target, the research coordinator may contact you to discuss any problems and help you adjust your plan safely.

**4.7 If You Miss a Session**

If you miss an exercise session because of illness, pain, bad weather, or another reason, record the reason in your diary. You may exercise on another day if you feel well enough, but do not exercise for an unusually long time or increase the intensity simply to make up the missed session.

**4.8 Adherence Target**

Please aim to complete at least 150 minutes of exercise each week and record the time you actually complete. If you are unable to reach the target, record the actual amount honestly. This will not automatically remove you from the study, and the research team can help you adjust the plan safely.

**4.9 Safety Precautions**

Stop exercising immediately and contact the research team or seek medical attention if you experience any of the following during or after exercise: chest pain or tightness, severe shortness of breath, dizziness or fainting, heart palpitations, severe joint pain or swelling, or any other symptoms that concern you. Do not exercise again until a doctor tells you that it is safe.

**Part 5: What Happens After the Week 16 Visit**

After your Week 16 visit, the first part of the study ends and the follow-up period begins. The follow-up period continues until Week 40. What you need to do during this period depends on your study group.

**For participants in the Integrated Strategy Group (ISG):**

**Stop:** You will stop taking Taodan granules after the Week 16 visit. You will no longer need to record Taodan granule use after that visit.

**Continue:** Keep taking fish oil at the same dose and continue your exercise plan until the Week 40 visit. Continue to aim for at least 150 minutes of moderate-intensity exercise each week, or follow the lower-impact plan agreed with your study doctor.

**Recording:** Continue to record your fish oil use and weekly exercise time after Week 16, using the Weekly Summary Log for Weeks 17–40 provided at the end of this handbook. Bring your fish oil packaging and ACVOR exercise records to the visits at Weeks 20, 24, 28, 32, 36, and 40. The research team will review these records with you at each visit.

**For participants in the Conventional Strategy Group (CSG):**

- You will continue your assigned conventional psoriasis treatment, including any treatment adjustment or additional skin treatment described in Section 1.3, as needed.
- You will not receive Taodan granules, fish oil supplementation, or the structured exercise programme from the study.
- You must continue to attend all follow-up visits (Weeks 20, 24, 28, 32, 36, and 40) and continue to report all psoriasis-related treatment changes as described in Section 1.2.

**Weekly Diary Log — Weeks 1 to 16**

Complete the diary each day as accurately as you can. Tick the boxes for the morning and evening doses you actually took. Record your exercise type and duration. You do not need to calculate the percentage of time spent in your target heart-rate range; the research team will obtain this from the wearable device or app. In the ‘Weekly cumulative exercise’ column, enter the total weekly exercise minutes on Day 7 (Sunday). The ‘Fish oil product checked at visit’ and ‘Staff verification’ columns will be completed by the research coordinator at your 4-weekly visits (Weeks 4, 8, 12, and 16); write N/A in these columns for non-visit weeks. If you miss a dose or do not exercise, leave the relevant box unticked and write the reason if possible. Do not take an extra dose or perform extra exercise simply to make up for a missed entry.

| **Week** | **Date(Mon/Day)** | **TDG taken(AM [ ] / PM [ ])** | **Fish oil taken(AM [ ] / PM [ ])** | **Exercisetype** | **Duration(min)** | **Heart rateduring exercise(bpm)** | **Time in target heart-rate range (from device/app)** | **Weekly cumulativeexercise (min)[fill on Day 7]** | **Fish oil productchecked at visit(Y/N/NA)** | **Staff verification(initials)** |
| --- | --- | --- | --- | --- | --- | --- | --- | --- | --- | --- |
| Week 1 | / | AM [ ] PM [ ] | AM [ ] PM [ ] |  |  |  |  |  | N/A | N/A |
|  | / | AM [ ] PM [ ] | AM [ ] PM [ ] |  |  |  |  |  | N/A | N/A |
|  | / | AM [ ] PM [ ] | AM [ ] PM [ ] |  |  |  |  |  | N/A | N/A |
|  | / | AM [ ] PM [ ] | AM [ ] PM [ ] |  |  |  |  |  | N/A | N/A |
|  | / | AM [ ] PM [ ] | AM [ ] PM [ ] |  |  |  |  |  | N/A | N/A |
|  | / | AM [ ] PM [ ] | AM [ ] PM [ ] |  |  |  |  |  | N/A | N/A |
|  | / | AM [ ] PM [ ] | AM [ ] PM [ ] |  |  |  |  |  |  |  |
| Week 2 | / | AM [ ] PM [ ] | AM [ ] PM [ ] |  |  |  |  |  | N/A | N/A |
|  | / | AM [ ] PM [ ] | AM [ ] PM [ ] |  |  |  |  |  | N/A | N/A |
|  | / | AM [ ] PM [ ] | AM [ ] PM [ ] |  |  |  |  |  | N/A | N/A |
|  | / | AM [ ] PM [ ] | AM [ ] PM [ ] |  |  |  |  |  | N/A | N/A |
|  | / | AM [ ] PM [ ] | AM [ ] PM [ ] |  |  |  |  |  | N/A | N/A |
|  | / | AM [ ] PM [ ] | AM [ ] PM [ ] |  |  |  |  |  | N/A | N/A |
|  | / | AM [ ] PM [ ] | AM [ ] PM [ ] |  |  |  |  |  |  |  |
| Week 3 | / | AM [ ] PM [ ] | AM [ ] PM [ ] |  |  |  |  |  | N/A | N/A |
|  | / | AM [ ] PM [ ] | AM [ ] PM [ ] |  |  |  |  |  | N/A | N/A |
|  | / | AM [ ] PM [ ] | AM [ ] PM [ ] |  |  |  |  |  | N/A | N/A |
|  | / | AM [ ] PM [ ] | AM [ ] PM [ ] |  |  |  |  |  | N/A | N/A |
|  | / | AM [ ] PM [ ] | AM [ ] PM [ ] |  |  |  |  |  | N/A | N/A |
|  | / | AM [ ] PM [ ] | AM [ ] PM [ ] |  |  |  |  |  | N/A | N/A |
|  | / | AM [ ] PM [ ] | AM [ ] PM [ ] |  |  |  |  |  |  |  |
| Week 4 | / | AM [ ] PM [ ] | AM [ ] PM [ ] |  |  |  |  |  | N/A | N/A |
|  | / | AM [ ] PM [ ] | AM [ ] PM [ ] |  |  |  |  |  | N/A | N/A |
|  | / | AM [ ] PM [ ] | AM [ ] PM [ ] |  |  |  |  |  | N/A | N/A |
|  | / | AM [ ] PM [ ] | AM [ ] PM [ ] |  |  |  |  |  | N/A | N/A |
|  | / | AM [ ] PM [ ] | AM [ ] PM [ ] |  |  |  |  |  | N/A | N/A |
|  | / | AM [ ] PM [ ] | AM [ ] PM [ ] |  |  |  |  |  | N/A | N/A |
|  | / | AM [ ] PM [ ] | AM [ ] PM [ ] |  |  |  |  |  |  |  |
| Week 5 | / | AM [ ] PM [ ] | AM [ ] PM [ ] |  |  |  |  |  | N/A | N/A |
|  | / | AM [ ] PM [ ] | AM [ ] PM [ ] |  |  |  |  |  | N/A | N/A |
|  | / | AM [ ] PM [ ] | AM [ ] PM [ ] |  |  |  |  |  | N/A | N/A |
|  | / | AM [ ] PM [ ] | AM [ ] PM [ ] |  |  |  |  |  | N/A | N/A |
|  | / | AM [ ] PM [ ] | AM [ ] PM [ ] |  |  |  |  |  | N/A | N/A |
|  | / | AM [ ] PM [ ] | AM [ ] PM [ ] |  |  |  |  |  | N/A | N/A |
|  | / | AM [ ] PM [ ] | AM [ ] PM [ ] |  |  |  |  |  |  |  |
| Week 6 | / | AM [ ] PM [ ] | AM [ ] PM [ ] |  |  |  |  |  | N/A | N/A |
|  | / | AM [ ] PM [ ] | AM [ ] PM [ ] |  |  |  |  |  | N/A | N/A |
|  | / | AM [ ] PM [ ] | AM [ ] PM [ ] |  |  |  |  |  | N/A | N/A |
|  | / | AM [ ] PM [ ] | AM [ ] PM [ ] |  |  |  |  |  | N/A | N/A |
|  | / | AM [ ] PM [ ] | AM [ ] PM [ ] |  |  |  |  |  | N/A | N/A |
|  | / | AM [ ] PM [ ] | AM [ ] PM [ ] |  |  |  |  |  | N/A | N/A |
|  | / | AM [ ] PM [ ] | AM [ ] PM [ ] |  |  |  |  |  |  |  |
| Week 7 | / | AM [ ] PM [ ] | AM [ ] PM [ ] |  |  |  |  |  | N/A | N/A |
|  | / | AM [ ] PM [ ] | AM [ ] PM [ ] |  |  |  |  |  | N/A | N/A |
|  | / | AM [ ] PM [ ] | AM [ ] PM [ ] |  |  |  |  |  | N/A | N/A |
|  | / | AM [ ] PM [ ] | AM [ ] PM [ ] |  |  |  |  |  | N/A | N/A |
|  | / | AM [ ] PM [ ] | AM [ ] PM [ ] |  |  |  |  |  | N/A | N/A |
|  | / | AM [ ] PM [ ] | AM [ ] PM [ ] |  |  |  |  |  | N/A | N/A |
|  | / | AM [ ] PM [ ] | AM [ ] PM [ ] |  |  |  |  |  |  |  |
| Week 8 | / | AM [ ] PM [ ] | AM [ ] PM [ ] |  |  |  |  |  | N/A | N/A |
|  | / | AM [ ] PM [ ] | AM [ ] PM [ ] |  |  |  |  |  | N/A | N/A |
|  | / | AM [ ] PM [ ] | AM [ ] PM [ ] |  |  |  |  |  | N/A | N/A |
|  | / | AM [ ] PM [ ] | AM [ ] PM [ ] |  |  |  |  |  | N/A | N/A |
|  | / | AM [ ] PM [ ] | AM [ ] PM [ ] |  |  |  |  |  | N/A | N/A |
|  | / | AM [ ] PM [ ] | AM [ ] PM [ ] |  |  |  |  |  | N/A | N/A |
|  | / | AM [ ] PM [ ] | AM [ ] PM [ ] |  |  |  |  |  |  |  |
| Week 9 | / | AM [ ] PM [ ] | AM [ ] PM [ ] |  |  |  |  |  | N/A | N/A |
|  | / | AM [ ] PM [ ] | AM [ ] PM [ ] |  |  |  |  |  | N/A | N/A |
|  | / | AM [ ] PM [ ] | AM [ ] PM [ ] |  |  |  |  |  | N/A | N/A |
|  | / | AM [ ] PM [ ] | AM [ ] PM [ ] |  |  |  |  |  | N/A | N/A |
|  | / | AM [ ] PM [ ] | AM [ ] PM [ ] |  |  |  |  |  | N/A | N/A |
|  | / | AM [ ] PM [ ] | AM [ ] PM [ ] |  |  |  |  |  | N/A | N/A |
|  | / | AM [ ] PM [ ] | AM [ ] PM [ ] |  |  |  |  |  |  |  |
| Week 10 | / | AM [ ] PM [ ] | AM [ ] PM [ ] |  |  |  |  |  | N/A | N/A |
|  | / | AM [ ] PM [ ] | AM [ ] PM [ ] |  |  |  |  |  | N/A | N/A |
|  | / | AM [ ] PM [ ] | AM [ ] PM [ ] |  |  |  |  |  | N/A | N/A |
|  | / | AM [ ] PM [ ] | AM [ ] PM [ ] |  |  |  |  |  | N/A | N/A |
|  | / | AM [ ] PM [ ] | AM [ ] PM [ ] |  |  |  |  |  | N/A | N/A |
|  | / | AM [ ] PM [ ] | AM [ ] PM [ ] |  |  |  |  |  | N/A | N/A |
|  | / | AM [ ] PM [ ] | AM [ ] PM [ ] |  |  |  |  |  |  |  |
| Week 11 | / | AM [ ] PM [ ] | AM [ ] PM [ ] |  |  |  |  |  | N/A | N/A |
|  | / | AM [ ] PM [ ] | AM [ ] PM [ ] |  |  |  |  |  | N/A | N/A |
|  | / | AM [ ] PM [ ] | AM [ ] PM [ ] |  |  |  |  |  | N/A | N/A |
|  | / | AM [ ] PM [ ] | AM [ ] PM [ ] |  |  |  |  |  | N/A | N/A |
|  | / | AM [ ] PM [ ] | AM [ ] PM [ ] |  |  |  |  |  | N/A | N/A |
|  | / | AM [ ] PM [ ] | AM [ ] PM [ ] |  |  |  |  |  | N/A | N/A |
|  | / | AM [ ] PM [ ] | AM [ ] PM [ ] |  |  |  |  |  |  |  |
| Week 12 | / | AM [ ] PM [ ] | AM [ ] PM [ ] |  |  |  |  |  | N/A | N/A |
|  | / | AM [ ] PM [ ] | AM [ ] PM [ ] |  |  |  |  |  | N/A | N/A |
|  | / | AM [ ] PM [ ] | AM [ ] PM [ ] |  |  |  |  |  | N/A | N/A |
|  | / | AM [ ] PM [ ] | AM [ ] PM [ ] |  |  |  |  |  | N/A | N/A |
|  | / | AM [ ] PM [ ] | AM [ ] PM [ ] |  |  |  |  |  | N/A | N/A |
|  | / | AM [ ] PM [ ] | AM [ ] PM [ ] |  |  |  |  |  | N/A | N/A |
|  | / | AM [ ] PM [ ] | AM [ ] PM [ ] |  |  |  |  |  |  |  |
| Week 13 | / | AM [ ] PM [ ] | AM [ ] PM [ ] |  |  |  |  |  | N/A | N/A |
|  | / | AM [ ] PM [ ] | AM [ ] PM [ ] |  |  |  |  |  | N/A | N/A |
|  | / | AM [ ] PM [ ] | AM [ ] PM [ ] |  |  |  |  |  | N/A | N/A |
|  | / | AM [ ] PM [ ] | AM [ ] PM [ ] |  |  |  |  |  | N/A | N/A |
|  | / | AM [ ] PM [ ] | AM [ ] PM [ ] |  |  |  |  |  | N/A | N/A |
|  | / | AM [ ] PM [ ] | AM [ ] PM [ ] |  |  |  |  |  | N/A | N/A |
|  | / | AM [ ] PM [ ] | AM [ ] PM [ ] |  |  |  |  |  |  |  |
| Week 14 | / | AM [ ] PM [ ] | AM [ ] PM [ ] |  |  |  |  |  | N/A | N/A |
|  | / | AM [ ] PM [ ] | AM [ ] PM [ ] |  |  |  |  |  | N/A | N/A |
|  | / | AM [ ] PM [ ] | AM [ ] PM [ ] |  |  |  |  |  | N/A | N/A |
|  | / | AM [ ] PM [ ] | AM [ ] PM [ ] |  |  |  |  |  | N/A | N/A |
|  | / | AM [ ] PM [ ] | AM [ ] PM [ ] |  |  |  |  |  | N/A | N/A |
|  | / | AM [ ] PM [ ] | AM [ ] PM [ ] |  |  |  |  |  | N/A | N/A |
|  | / | AM [ ] PM [ ] | AM [ ] PM [ ] |  |  |  |  |  |  |  |
| Week 15 | / | AM [ ] PM [ ] | AM [ ] PM [ ] |  |  |  |  |  | N/A | N/A |
|  | / | AM [ ] PM [ ] | AM [ ] PM [ ] |  |  |  |  |  | N/A | N/A |
|  | / | AM [ ] PM [ ] | AM [ ] PM [ ] |  |  |  |  |  | N/A | N/A |
|  | / | AM [ ] PM [ ] | AM [ ] PM [ ] |  |  |  |  |  | N/A | N/A |
|  | / | AM [ ] PM [ ] | AM [ ] PM [ ] |  |  |  |  |  | N/A | N/A |
|  | / | AM [ ] PM [ ] | AM [ ] PM [ ] |  |  |  |  |  | N/A | N/A |
|  | / | AM [ ] PM [ ] | AM [ ] PM [ ] |  |  |  |  |  |  |  |
| Week 16 | / | AM [ ] PM [ ] | AM [ ] PM [ ] |  |  |  |  |  | N/A | N/A |
|  | / | AM [ ] PM [ ] | AM [ ] PM [ ] |  |  |  |  |  | N/A | N/A |
|  | / | AM [ ] PM [ ] | AM [ ] PM [ ] |  |  |  |  |  | N/A | N/A |
|  | / | AM [ ] PM [ ] | AM [ ] PM [ ] |  |  |  |  |  | N/A | N/A |
|  | / | AM [ ] PM [ ] | AM [ ] PM [ ] |  |  |  |  |  | N/A | N/A |
|  | / | AM [ ] PM [ ] | AM [ ] PM [ ] |  |  |  |  |  | N/A | N/A |
|  | / | AM [ ] PM [ ] | AM [ ] PM [ ] |  |  |  |  |  |  |  |

**Weekly Summary Log — Weeks 17 to 40**

This simplified log covers the Week 16–40 follow-up period, when participants in the Integrated Strategy Group continue fish oil and exercise after stopping Taodan granules. Please record your fish oil intake and total weekly exercise minutes each week. Bring this log, your fish oil packaging, and your ACVOR device records to your visits at Weeks 20, 24, 28, 32, 36, and 40, where the research team will review them with you and complete the shaded columns. Write N/A in the visit-related columns for weeks without a scheduled visit.

| **Week** | **Fish oil — mornings taken (out of 7)** | **Fish oil — evenings taken (out of 7)** | **Total exercise minutes this week** | **Device data reviewed (Y/N/NA)** | **Fish oil product checked (Y/N/NA)** | **Staff verification (initials)** |
| --- | --- | --- | --- | --- | --- | --- |
| Week 17 |  |  |  | N/A | N/A | N/A |
| Week 18 |  |  |  | N/A | N/A | N/A |
| Week 19 |  |  |  | N/A | N/A | N/A |
| Week 20 |  |  |  |  |  |  |
| Week 21 |  |  |  | N/A | N/A | N/A |
| Week 22 |  |  |  | N/A | N/A | N/A |
| Week 23 |  |  |  | N/A | N/A | N/A |
| Week 24 |  |  |  |  |  |  |
| Week 25 |  |  |  | N/A | N/A | N/A |
| Week 26 |  |  |  | N/A | N/A | N/A |
| Week 27 |  |  |  | N/A | N/A | N/A |
| Week 28 |  |  |  |  |  |  |
| Week 29 |  |  |  | N/A | N/A | N/A |
| Week 30 |  |  |  | N/A | N/A | N/A |
| Week 31 |  |  |  | N/A | N/A | N/A |
| Week 32 |  |  |  |  |  |  |
| Week 33 |  |  |  | N/A | N/A | N/A |
| Week 34 |  |  |  | N/A | N/A | N/A |
| Week 35 |  |  |  | N/A | N/A | N/A |
| Week 36 |  |  |  |  |  |  |
| Week 37 |  |  |  | N/A | N/A | N/A |
| Week 38 |  |  |  | N/A | N/A | N/A |
| Week 39 |  |  |  | N/A | N/A | N/A |
| Week 40 |  |  |  |  |  |  |
