# Supplementary material for: Protocol for a cohort study of comprehensive intervention of psoriasis and diabetes comorbidity prevention and control using traditional Chinese medicine of blood-activating and stasis-dissolving method
Source: Front Med (Lausanne). 2026 Oct 14;13:1831495. doi: 10.3389/fmed.2026.1831495 (PMC13717394; doi:10.3389/fmed.2026.1831495)
Supplement: Supplementary file 3 [file Supplementary_File_3.docx]

**Supplementary file 3. Serious Adverse Event Report Form**

| Report Type | ☐ Initial Report ☐ Follow-up Report   ☐ Final Report | | | Report Date: | |
| --- | --- | --- | --- | --- | --- |
| Medical Institution & Specialty | Name: ________________________ | | | Tel: ______________ | |
| Sponsor Name | Name: ________________________ | | | Tel: | |
| Relationship between SAE and each intervention | | | | | |
|  | Definitely related | Possibly related | Probably related | | Unrelated |
| TDG | ☐ | ☐ | ☐ | | ☐ |
| Fish oil | ☐ | ☐ | ☐ | | ☐ |
| Exercise | ☐ | ☐ | ☐ | | ☐ |
| Subject Basic Information | Subject ID: ________________________  Date of Birth: ________________________  Gender: ☐ Male ☐ Female  Height (cm): ________________________  Weight (kg): ________________________  Ethnicity: ________________________  Age at SAE Onset: ________________________  Concomitant Diseases and Treatments: ☐ Yes ☐ No | | | | |
| Medical Term (Diagnosis) of SAE | ________________________________________________________ | | | | |
| SAE Details | ☐ Death Date: ______ / ____ / ____ ☐ Resulting in hospitalization  ☐ Prolonged hospitalization ☐ Disability ☐ Functional disorder  ☐ Induced congenital malformation ☐ Life-threatening ☐ Others: _______________ | | | | |
|  |  | | | | |
| Time Points | Date of SAE Onset: ________________________ | | | Date Investigator was informed of SAE: ___________ | |
| Measures for Intervention | Measures taken for suspected intervention(s): ☐ Continue ☐ Dose reduction ☐ Suspended then resumed ☐ Discontinue ☐ Not applicable ☐ Unknown ☐ Other (specify) | | | | |
| Outcome of SAE | ☐ Symptoms resolved (Sequelae: ☐ Yes ☐ No) ☐ Improved ☐ Not improved ☐ Death ☐ Unknown | | | | |
| SAE Reporting History | Reported domestically: ☐ Yes ☐ No ☐ Unknown Reported internationally: ☐ Yes ☐ No ☐ Unknown | | | | |

Reporting Institution: ________________________

Reporter’s Title / Professional Title: _______________

Tel: ________________________

Reporter’s Signature & Date: _____________________

SAE, Serious Adverse Event; TDG, Taodan granules.
