# Supplementary material for: Protocol for a cohort study of comprehensive intervention of psoriasis and diabetes comorbidity prevention and control using traditional Chinese medicine of blood-activating and stasis-dissolving method
Source: Front Med (Lausanne). 2026 Oct 14;13:1831495. doi: 10.3389/fmed.2026.1831495 (PMC13717394; doi:10.3389/fmed.2026.1831495)
Supplement: Supplementary file 4 [file Supplementary_File_4.docx]

**Supplementary File 4. Statistical Analysis Plan**

Protocol for a Cohort Study of Comprehensive Intervention of Psoriasis and Diabetes Comorbidity Prevention and Control Using Traditional Chinese Medicine of Blood-Activating and Stasis-Dissolving Method

**1. General Considerations**

This document describes the statistical analysis methods for the above-named study. All analyses will be performed using R (Version 4.4.3). The principal investigator and a qualified statistician will be responsible for the analysis. The analysis will follow the principles described in the main manuscript (Section 2.9).

The core estimands, analysis sets, covariates, matching specifications, intercurrent-event strategies, and sensitivity analyses are prespecified in this document. Any remaining operational details that do not change these prespecified principles will be finalised in a version-controlled final SAP before database lock and before access to treatment-group outcome comparisons. Any subsequent change will be dated, justified, and reported as an SAP amendment.

**2. Analysis Sets**

Full Analysis Set (FAS): All enrolled participants who have received at least one administration of the assigned strategy (for ISG: at least one dose of TDG or fish oil, or initiation of the exercise programme; for CSG: at least one administration of the assigned conventional therapy) and have at least one post-baseline assessment of the outcome. This set follows the intention-to-treat principle and will be used for the primary efficacy analysis.

Safety Set (SS): All enrolled participants who have received at least one administration of the assigned strategy (as defined above) and have at least one post-baseline safety assessment. This set will be used for all safety analyses.

Per-Protocol Set (PPS): For the Week 16 primary-outcome sensitivity analysis, the PPS will comprise participants in the FAS who have a Week 16 primary-outcome assessment, have no major protocol deviation that materially affects eligibility, treatment exposure, or ascertainment of the primary outcome, and meet the prespecified adherence requirements during Weeks 1-16. For the ISG, adequate adherence requires receipt of at least 80% of the scheduled TDG doses, receipt of at least 80% of the scheduled fish oil doses, and achievement of the prescribed weekly exercise target in at least 80% of the prescribed Weeks 1-16. Exercise adherence during Weeks 1-4 will be assessed against the individualised weekly ramp-up target. For the CSG, adherence to the assigned conventional-treatment strategy and the absence of a major treatment-related deviation will be required. Events occurring after Week 16 will not determine PPS eligibility for the Week 16 primary-outcome analysis.

**3. Sample Size Calculation**

The sample size was estimated based on a two-independent-sample mean comparison for the primary outcome (change in HOMA-IR from baseline to Week 16). Based on published data (Melita et al., 2018), the mean HOMA-IR in patients with severe psoriasis was 4.13 (SD 3.75). The intervention group was expected to achieve a mean HOMA-IR of 2.93, representing a clinically meaningful improvement. The anticipated mean difference between groups was 1.20. To account for greater population heterogeneity, the pooled standard deviation was conservatively set at 3.20.

With a two-sided α=0.05 and power =0.80, the required sample size per group was calculated using the formula:

where Z_α/2_=1.96, Z_β_=0.84, σ=3.20, and δ=1.20. The calculation yields n≈112 per group.

Accounting for a 20% dropout rate common in lifestyle-intervention trials, the final recruitment target was set at 140 participants per group, for a total of 280 participants.

**4. Primary Efficacy Analysis**

Primary Outcome: Change from baseline in HOMA-IR at Week 16.

Primary Confirmatory Contrast: The primary confirmatory analysis is the between-group difference in the mean change in HOMA-IR from baseline to Week 16 (ISG vs. CSG), estimated from the model below. This Week 16 contrast is the sole confirmatory test for the primary outcome.

Statistical Model: A linear mixed-effects model (LMM) with treatment group (CSG vs. ISG), time (Weeks 0, 16, 40), Group × Time interaction, and the prespecified covariate set (see Section 7) as fixed effects, and a random intercept for each participant. The Group × Time interaction evaluated across all three time points serves as a secondary/sensitivity analysis of the longitudinal trajectory and does not supersede the prespecified Week 16 primary contrast above.

Exercise adherence and exposure: Post-baseline exercise exposure will not be included as an adjustment covariate in the primary confirmatory model because exercise is a component of the integrated strategy and may lie on the pathway through which the strategy affects the outcome. Exercise adherence will be classified using the prespecified weekly targets and summarised by study group. Exercise exposure may be examined in descriptive and exploratory analyses but will not alter the primary estimand.

Stratification: The model will be stratified by the class of conventional therapy (biologics vs. small-molecule inhibitors).

Sensitivity Analyses: Will include multiple imputation for missing data, complete case analysis, analysis on the Per-Protocol Set (PPS), and propensity score matching (PSM) as described in Section 6.

**5. Secondary and Exploratory Analyses**

Secondary Outcomes (change in HOMA-IR at Week 40, PASI, lipid parameters, etc.) and exploratory outcomes (T2DM incidence, inflammatory biomarkers) will be analyzed using similar LMMs or logistic regression as appropriate.

Biomarker Assay Reporting: In the final trial report, each exploratory biomarker assay will be documented with the manufacturer, catalogue number, lot number, limit of detection, lower limit of quantification, intra-assay coefficient of variation, inter-assay coefficient of variation, and all prespecified quality-control results.

Multiplicity: The primary outcome is confirmatory. All secondary and exploratory outcomes will be reported descriptively or as hypothesis-generating, without formal multiplicity adjustment.

**6. Propensity Score Matching (Sensitivity Analysis)**

To mitigate selection bias, 1:1 nearest-neighbour matching without replacement will be performed within each stratum of conventional therapy class (biologics vs. small-molecule inhibitors), using a fixed caliper of 0.20 standard deviations of the logit of the estimated propensity score. The propensity-score model will include the following prespecified baseline covariates: age, sex, BMI, baseline PASI score, disease duration, baseline HOMA-IR, baseline fasting plasma glucose, baseline lipid parameters (TC, TG, HDL-C, LDL-C), prior psoriasis treatment history (topical therapy only vs. systemic therapy/biologics), and the T2DM risk score derived from the previously validated multifactorial model (Gao et al., 2024). No covariate will be selected or removed on the basis of statistical significance or the observed outcome. Unmatched participants will be excluded only from this PSM sensitivity analysis and will remain in the primary FAS analysis. Covariate balance will be acceptable only if the absolute standardised mean difference is <0.10 for every prespecified covariate. If this criterion is not achieved, the caliper will not be changed after inspection of the data; the imbalance and the number of unmatched participants will be reported, the PSM result will be labelled as not adequately balanced and interpreted as exploratory, and the primary FAS result will remain the basis for inference. The matched outcome analysis will use the primary LMM with random intercepts for matched pair and participant to account for dependence within matched pairs and repeated measurements within participants. Variance inflation factors will be reported as a diagnostic; continuous covariates may be centred to improve numerical stability, but no prespecified covariate will be removed solely because of its VIF. No inverse-probability-weighted analysis is planned.

**7. Covariate Sets**

The prespecified covariate set for the primary LMM and PSM includes: age, sex, BMI, baseline PASI score, disease duration, baseline HOMA-IR, baseline fasting plasma glucose, baseline lipid parameters (TC, TG, HDL-C, LDL-C), prior psoriasis treatment history, and the T2DM risk score.

**8. Missing Data Handling**

For the primary outcome in the FAS, multiple imputation under the missing-at-random assumption will be used. Complete case analysis will be performed as a sensitivity analysis.

**9. Treatment Modifications, Rescue Therapy, and Protocol Deviations**

Operational treatment-modification rule: Participants who have not achieved PASI 50 at the Week 16 visit may undergo treatment intensification within the same permitted therapeutic class. The intensified regimen will be maintained from Week 16 until the Week 28 reassessment, unless a change is required for safety or another clinically compelling reason. Response will be formally reassessed at the Week 28 visit. Protocol-permitted topical rescue therapy may be initiated if PASI 50 has still not been achieved. Treatment intensification, dose or frequency changes, switching, rescue therapy, and any earlier medically necessary treatment will be recorded with the date, visit, reason, and relevant treatment details.

Primary estimand strategy: The primary FAS analysis will use a treatment-policy strategy. All observed outcome measurements will be analysed according to the assigned study strategy, irrespective of treatment escalation, treatment switching, permitted dose changes, rescue therapy, treatment discontinuation, or minor or major protocol deviations. These events will not be treated as missing solely because they occurred.

Event recording and summaries: Treatment escalation, switching, dose changes, rescue therapy, and protocol deviations will be recorded with the event type, date, visit, reason, and relevant treatment details. Counts, proportions, timing, and reasons will be summarised by study group. Permitted topical retinoids will be recorded as concomitant treatment; systemic retinoid use will be classified as prohibited treatment and a major protocol deviation.

Protocol deviations: Minor deviations will not exclude a participant or observation from the FAS or PPS. Major deviations will not exclude data from the primary FAS analysis. Before database lock and without access to treatment-group outcome comparisons, each deviation will be classified as minor or major using the deviation-classification rules documented in the version-controlled final SAP, and the classification will be recorded in a blinded deviation review listing. The PPS sensitivity analysis will exclude participants with major deviations that materially affect exposure, eligibility, or ascertainment of the primary outcome; the number and reasons for exclusions will be reported by group.

Sensitivity analysis for intercurrent events: A supplementary while-on-treatment analysis will censor outcome measurements obtained after the first treatment switch, prohibited systemic retinoid use, rescue therapy, or other major treatment-related deviation. Missing post-event Week 16 outcomes will be multiply imputed under the missing-at-random assumption using treatment group, the prespecified baseline covariates, pre-event outcome measurements, and the type and timing of the intercurrent event. Results will be compared with the treatment-policy FAS analysis. This sensitivity analysis will not replace the primary estimand.

**10. Safety Analyses**

All safety analyses will be performed on the Safety Set (SS). This includes summaries of adverse events (AEs) by group, laboratory parameters, vital signs, and protocol deviations, as described in the main manuscript (Sections 2.6 and 2.8).

**11. Software**

All analyses will be performed using R (Version 4.4.3). Analysis code will be archived and made available upon reasonable request.
